# Supplementary material for: The 20-year impact of tobacco price and tobacco control expenditure increases in Minnesota, 1998-2017
Source: PLoS One. 2020 Mar 18;15(3):e0230364. doi: 10.1371/journal.pone.0230364 (PMC7080278; doi:10.1371/journal.pone.0230364)
Supplement: S1 Supplement — (DOCX) [file pone.0230364.s001.docx]

**Supplement S1**

HealthPartners Institute

ModelHealth^TM^: Tobacco MN

Model documentation

Model Version 1.2

September 28, 2019

Table of Contents

[Introduction 4](#_Toc21265803)

[Model structure 4](#_Toc21265804)

[Overview 4](#_Toc21265805)

[Software 5](#_Toc21265806)

[Model Initialization, population characteristics 5](#_Toc21265807)

[The smoking behavior module 6](#_Toc21265808)

[Initial smoking status 7](#_Toc21265809)

[Estimating initial smoking status 7](#_Toc21265810)

[Assigning age of smoking initiation and cessation 8](#_Toc21265811)

[Estimating changes in smoking status 9](#_Toc21265812)

[Relapse rates 12](#_Toc21265813)

[The Health Impact Module 13](#_Toc21265814)

[SAMMEC-based disease and burden estimation 13](#_Toc21265815)

[Smoking-attributable mortality risk by age, sex and smoking status 13](#_Toc21265816)

[Smoking-attributable disease risk by age, sex and smoking status 14](#_Toc21265817)

[Smoking-attributable diseases, health utilities, and duration 14](#_Toc21265818)

[Competing causes of death 16](#_Toc21265819)

[Costs and productivity 16](#_Toc21265820)

[Smoking-attributable medical costs 16](#_Toc21265821)

[Productivity 19](#_Toc21265822)

[References 21](#_Toc21265823)

[Supplemental Tables 24](#_Toc21265824)

[Coefficients estimated for the health insurance submodel 24](#_Toc21265825)

[Initial smoking status at time 0 (at model initiation) 29](#_Toc21265826)

[Incident smoking-attributable disease cases and mortality rates 33](#_Toc21265827)

ModelHealth:Tobacco MN

# Introduction

The HealthPartners Institute ModelHealth^TM^:Tobacco MN was developed to evaluate the health impact and cost-effectiveness of observed reductions in the prevalence of cigarette smoking in Minnesota and to estimate the impact of Minnesota tobacco control policy. ModelHealth: Tobacco MN was constructed by modifying the original US version, ModelHealth: Tobacco.[^1^](#_ENREF_1)^,^[^2^](#_ENREF_2)

ModelHealth:Tobacco MN estimates behavioral, health, and medical utilization and productivity impact of tobacco control programs and policy. The model employs a flexible microsimulation framework in which individuals, rather than population groups, are simulated over time. Individual behaviors and outcomes are simulated and recorded each year, and the experiences of simulated individuals are aggregated up to the community level to estimate population health and economic impact.

The model starts with a simulated population that is representative of the Minnesota population with respect to age, sex, race-ethnicity and educational status. Over time, youth who are representative of the sex and race-ethnicity distribution of recent birth cohorts age into the model.

This document provides an overview of the base model’s structure, the development of the inputs to the base model, and discussion of the modeling framework and embedded algorithms. Inputs specific to clinical interventions, policies and programs (counseling, tobacco taxes, media campaigns etc.) are discussed in manuscripts or other reports specific to their analysis.

# Model structure

## Overview

ModelHealth: Tobacco MN is a Markovian individual-based simulation model (i.e. Markov microsimulation). In the health care context, a Markov microsimulation is a model in which simulated individuals age over time, while facing period-specific probabilities (‘risks’) of changing health behaviors and experiencing related health outcomes and economic impact. In each cycle (cycle length is one year in ModelHealth: Tobacco), individuals age and either remain in their current state (smoking status, health status, etc.) or they transition to a different state. Transition probabilities are obtained from literature or analyses of relevant data sets. In the model, the state of each individual is tracked over time, producing a complete year-by-year history of all model outcomes.

The model can be conceptualized as having the three distinct parts, as shown in Figure S1.1 and described below, plus an insurance sub-model that is integrated into each of these parts. The first part, Model Initiation, defines the population to be modeled. The second part, the Smoking Behavior Module, determines transitions in smoking status over time, and the third part, the Health Effects Module, determines health and economic outcomes associated with cigarette smoking, which includes smoking-attributable (SA) medical costs and productivity losses.

**Figure S1.1. Structure of the Smoking Prevention Microsimulation Model**


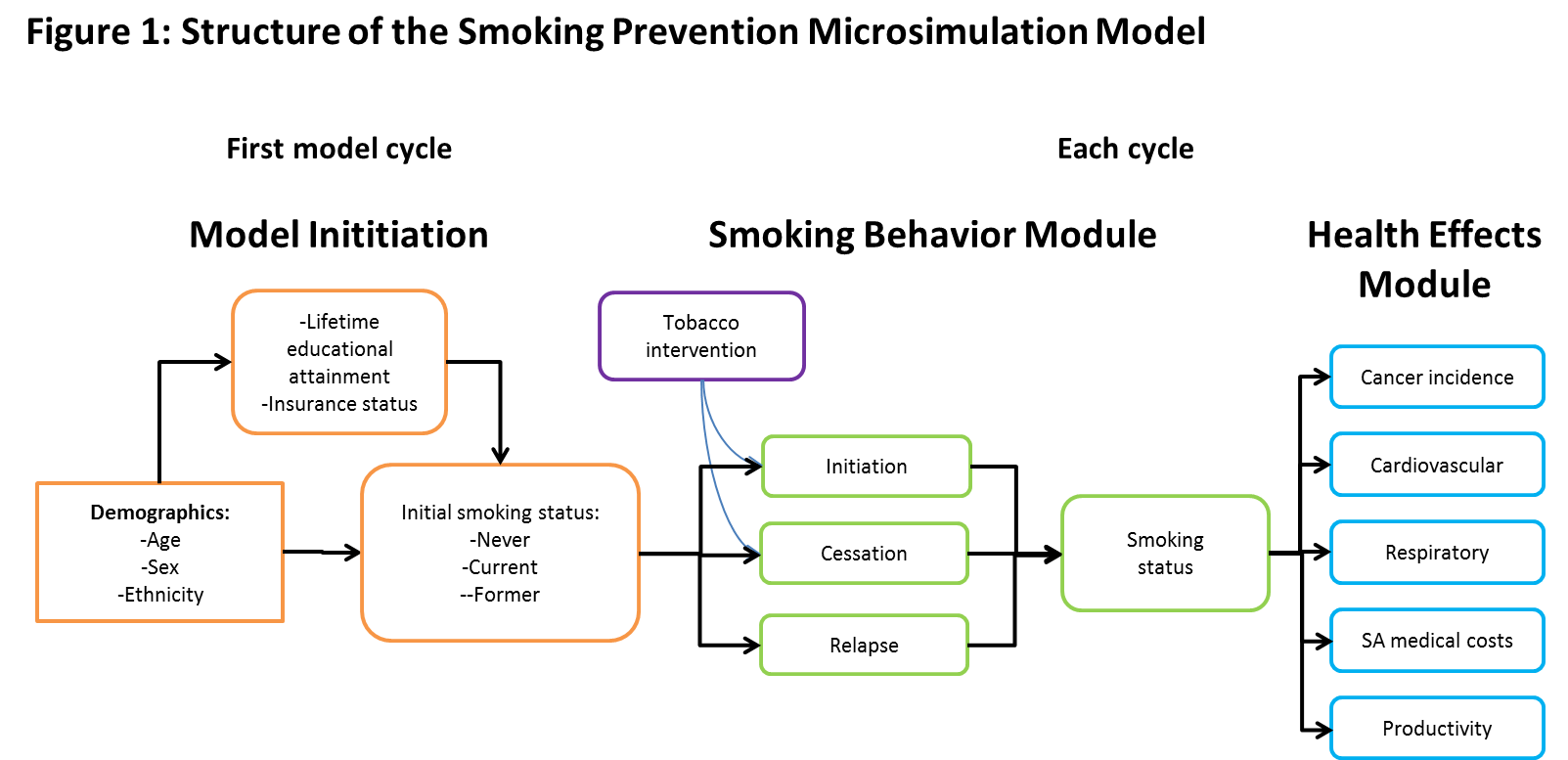


## Software

The model is programmed in TreeAge PRO 2015. The structure of the model reflects some of the capabilities and limitations of TreeAge PRO with respect to microsimulation modeling. Within TreeAge PRO, the model uses multiple custom Python functions in both the base model and in implementing programs and policies for analyses. Outside of TreeAge PRO, we employ Java to import some model inputs. We process model results in R and Microsoft Excel to summarize population-level results.

# Model Initialization, population characteristics

ModelHealth: Tobacco MN starts by generating a population of heterogeneous simulated individuals, or agents. Four broad racial/ethnic groups are currently represented: black, Hispanic, white, and other. An agent’s lifetime educational achievement at age 25 is determined based on sex and race-ethnicity. Three broad levels of lifetime educational achievement are contained in the model: no high school diploma, high school degree with or without additional years of education with less than a bachelor’s degree, and bachelor’s degree or higher. Broad categories of race/ethnicity and educational status were used because they are consistently defined and identifiable across the multiple data sources used to parameterize the model.

To allow projections of population impact in future years, cohorts of individuals who are not yet alive are defined at model initiation and they are “born into” the model over time. At model initiation, these cohorts are represented with negative ages. They then age into the analysis age range as the model runs. For example, cohorts with an initial age of -5 represent a future birth cohort that will be born in year 5 of the simulation. As the simulation progresses, the size of the model population grows over time because young cohorts are introduced in numbers that represent their larger size compared to the older pre- World War cohorts who they replace. The model does not incorporate projections of net migration. However, results from the simulated population size are scaled to reflect the actual size of the Minnesota population.

The model’s baseline year is 1997. We populated the model with simulated individuals representative of the Minnesota population in 1997 in terms of age, sex, race/ethnicity, and educational attainment using probabilities derived from the U.S. Current Population Survey.[^3^](#_ENREF_3) Simulated individuals who are added to the model over time are representative of Minnesota youth in 1997.

Simulated individuals are also assigned disability, employment and poverty status. These characteristics partially determine the primary type of insurance status. The demographics of the Minnesota population were associated with employment status, poverty status, disability status and health insurance type using relationships between demographics and these characteristics that we estimated for the United States as a whole from the Public Use Microdata Series of the U.S. Current Population Survey[^4^](#_ENREF_4) and the Survey of Income and Program Participation.[^5^](#_ENREF_5) The beta coefficients from logistic regressions are provided in tables at the end of this supplement.

# The smoking behavior module

The model tracks smoking behavior in each year of a simulation and uses the smoking behavior module and the health effects module to determine the disease risk and health outcomes associated with that behavior. If an agent’s smoking status changes due to introduction of a policy in the model or an exogenous recalibration of initiation and cessation rates, his or her health outcomes may or may not change. For example, some youth who would have started smoking without a tobacco tax increase will remain non-smokers with a tax increase. Of those who remain non-smokers as youth with the tax increase, some may still start smoking as young adults while others avoid a lifetime of tobacco use. Some smokers never experience significant harms of smoking; some by chance and others by quitting in time to reduce their risks. For those smokers who would never experience harm, avoiding smoking initiation has no impact on health outcomes. Others who avoid smoking due to the tax increase will avoid smoking-attributable disease and may have significantly longer lives. Whether a policy change or exogenous change to initiation or cessation impacts a particular smoker’s health depends on what would have happened to that individual without the change and how he or she responds to the change. Through a series of evidence-informed probabilities, the microsimulation produces and tracks these heterogeneous individual experiences. We calculate the population-wide impact by summing these experiences.

## Initial smoking status

Adults may be in one of three smoking states: *never smoker*, *current smoker* and *former smoker*. Youth (younger than age 18), may be never or current smokers. Cessation and status as former smokers are not tracked for youth in the model due to the experimental nature of youth smoking and associated limitations of the data that quantify youth smoking.

We approximated the associations between demographics and smoking status of youth in 1997 using de-identified Minnesota Youth Tobacco Survey (MYTS) responses provided by the Minnesota Department of Health from the first MYTS survey in 2000.[^6^](#_ENREF_6) Using year 2000 prevalence rates likely produces a conservative baseline estimate of youth smoking in 1997, as national rates for youth were trending slightly downward in the late 1990s.[^7^](#_ENREF_7) MYTS data include middle-school and high-school students. Age trends were extrapolated to derive probabilities of current smoking and net initiation back to age 9 with a simplifying assumption that smoking prevalence is zero at age 8.

Adult smoking status is defined using the usual criteria of ever having smoked 100 cigarettes:

- **Never smoker:** Having smoked fewer than 100 cigarettes in their lifetime
- **Current smoker:** Having smoked at least 100 cigarettes in their lifetime and having smoked in the last week
- **Former smoker:** Having smoked at least 100 cigarettes in their lifetime and not currently a smoker

We estimated adult cigarette smoking status in 1997 from self-report of the Minnesotans who responded to the 1996 or 1997 Behavioral Risk Factor Surveillance Surveys (BRFSS).[^8^](#_ENREF_8) For ages 65 and older, we calibrated the initial smoking prevalence derived from BRFSS to be consistent with that from the first Minnesota Adult Tobacco Survey (MATS) in 1999.[^9^](#_ENREF_9) This was done to create internal consistency with the Realized Prevalence Scenario that is used in some analyses.

At model initiation, the likelihood that an agent is in any one of the three smoking states is conditioned on his/her age, gender, and – for those older than age 25 – the lifetime educational attainment at introduction into the model. Available survey samples for Minnesota are too small to allow accurate estimates of smoking status based on race-ethnicity. The likelihood that an agent currently in the never smoker state begins smoking within a given cycle is conditioned upon his/her age, gender, and – if older than age 25 – lifetime educational attainment. Our model specification intends no causal inference regarding the relationship between smoking behavior and educational attainment, merely an association.

### Estimating initial smoking status

A multinomial logistic regression predicting the three smoking states was used to estimate the likelihood of an adults having an initial smoking status given his or her age, sex, and lifetime educational attainment. (Table S1.1). The estimated distribution across potential smoking states was then used to determine each agent’s initial smoking status at introduction into the model. The statistical relationships between each covariate and other predictors were screened prior to inclusion of the covariate in a final risk equation. If the inclusion of a covariate violated assumptions (e.g., co-linearity, normality, disproportionate cell size) appropriate adjustments (e.g., center around mean, transformation, re-categorization) were made or its inclusion reconsidered. Probabilities of current and former smoking by age, sex and race-ethnicity are provided in tables at the end of this supplement.

| **Table S1.1. Multinomial logistic regression coefficients for adult initial smoking status** | | |
| --- | --- | --- |
|  | **Current smokers** | **Former smokers** |
| **Ages 18-24** | | |
| Intercept | -0.2386 | -1.6589 |
| Age^1^ | -0.0758 | -0.0413 |
| Female | -0.6755 | -2.2038 |
| Age^1^ x Female | 0.124 | 0.6333 |
| **Ages 25+** | | |
| Intercept | 1.855 | 0.1059 |
| Age^2^ | -0.0627 | 0.0127 |
| Female | -0.8063 | -0.5503 |
| High school graduate | -1.8112 | -1.3636 |
| College graduate | -3.5086 | -1.9054 |
| Age^2^ x Female | -0.005 | -0.029 |
| Age^2^ x High school graduate | 0.0343 | 0.0258 |
| Age^2^ x College graduate | 0.0651 | 0.038 |
| Female and High school graduate | 0.6862 | 0.9105 |
| Female and college graduate | 0.4885 | 0.6847 |
| ^1^Age less 18 years. ^2^Age less 25 years | | |

### Assigning age of smoking initiation and cessation

For agents who start the model as never smokers and become smokers, their age at smoking initiation is set at the time of initiation. Similarly, agents who are initialized as current smokers or become smokers during a simulation have their age at quit set at that time. An age of smoking initiation is assigned to all agents initialized as either a current smoker or a former smoker. In addition, an age of cessation is assigned for those initialized as a former smoker.

**Figure S1.2. Determining age of initiation and cessation**

Figure S1.2 depicts the process for an example agent initialized into the model as a 45-year old former smoker. The figure depicts hypothetical annual initiation and cessation rates without incorporating long-term relapse, and therefore it does not by itself determine change in prevalence over time for 45 year olds in the model. First, in Step 1, a random draw determines the age at which a current or former smoker first started smoking (age 19 in the example of Figure S1.2). That draw is from a distribution configured to initiation rates estimated from the National Health Interview Survey (NHIS). Then, for those initialized as former smokers (Step 2), a random draw determines the age of cessation (age 26). That draw is from a second distribution configured to cessation rates estimated from NHIS and truncated at the age of initiation. These two ages are used to determine the time spent smoking and time since quit. Time since quit is used in the model to assign the probability of relapse to each former smoker in each year, the year in which disease risks are reduced from current smoker risks to former smoker risks, and to calculate the portion of excess disease and productivity costs of smoking incurred by a former smoker each year.

## Estimating changes in smoking status

As the model runs, an individual’s “risk” of changing smoking status (i.e. *transitioning* *to another smoking state*), is determined by his or her current smoking state, demographics and, for current smokers, insurance status. The specification of changes in smoking status as described below, create a model baseline that predicts what would have occurred had smoking initiation and cessation rates been held constant at 1997 levels. ***Therefore, the baseline prediction does not incorporate program, policy, or cultural trends that have influenced initiation and cessation rates since 1997.***

Individuals who have never smoked can either remain in the never smoker state or begin smoking and transition to the current smoker state. A current smoker can remain in the current smoker state or quit and transition to the former smoker state. The utilization of smoking cessation medication by adults varies by insurance status. Therefore, insurance status influences the probability of adult cessation. A former smoker either relapses into the current smoker state or remains in the former smoker state. In addition, all individuals are faced with a risk of dying of either a tobacco-related illness or some other cause. Figure S1.3 illustrates this conceptual framework of the natural history of smoking.

**Figure S1.3. Natural History of Cigarette Smoking**


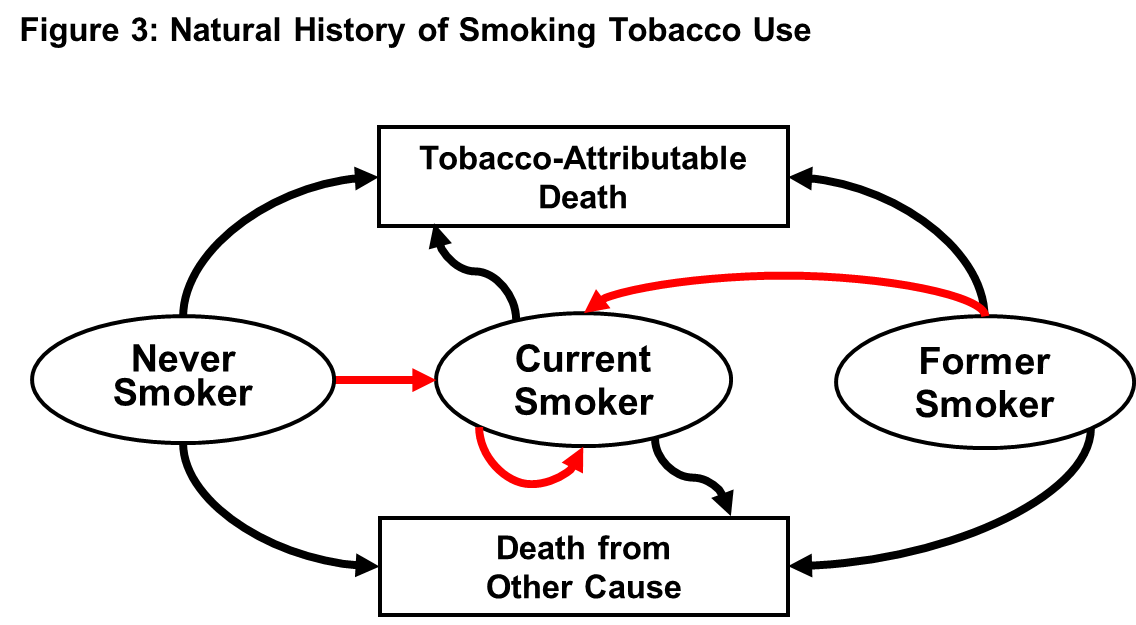


Youth who start smoking are presumed to remain smokers until they reach age 18. Non-smoking youth have a probability of initiating smoking. That probability is estimated to reflect the probability of initiating smoking, less cessation that does occur during that year of age. This estimate of “net initiation” allows accurate simulation of the prevalence of youth smoking from available data, but does not track former smoking status for youth. Net initiation for youth was estimated from MYTS.[^6^](#_ENREF_6)

Too few Minnesotans ages 18-24 were represented in available data sets to yield reliable estimates of cigarette smoking status, initiation rates and cessation rates. Therefore, we applied a simplifying assumption that cigarette smoking status from ages 18 to 24 was the same as the model’s predicted rates for 25-year-olds. These rates are also similar to predicted rates for 17-year-olds. Therefore, in effect, the model uses a peak lifetime tobacco prevalence that is nearly constant from ages 17 to 25. This age range is decades away from the years of peak risk for smoking-attributable disease. Therefore, this simplification has little impact on estimates of the health and economic impacts of tobacco use during the next 40 years. The net initiation rates that produce this prevalence pattern are shown in Table S1.2.

| **Table S1.2. Youth and young adult net initiation rates** | | |
| --- | --- | --- |
| **Age** | **Male** | **Female** |
| 9 | 0.004 | 0.006 |
| 10 | 0.010 | 0.006 |
| 11 | 0.010 | 0.011 |
| 12 | 0.018 | 0.027 |
| 13 | 0.063 | 0.063 |
| 14 | 0.066 | 0.066 |
| 15 | 0.088 | 0.102 |
| 16 | 0.075 | 0.072 |
| 17 | 0.038 | 0.047 |
| 18-24 | 0.024 | 0.024 |

For adult smokers older than 25, we estimated cessation rates from combined 1996 and 1997 BRFSS responses.[^8^](#_ENREF_8) The coefficients from the regression are shown in Table S1.3. We assumed the probability of smoking initiation after age 25 is zero.

| **Table S1.3. Logistic regression coefficients for smoking cessation** | |
| --- | --- |
| **Variable** | **Coefficient** |
| Intercept | -3.0892 |
| Age minus 25 | 0.0165 |
| Female | 0.5211 |
| High School Education (w/out 4-year degree) | 0.7947 |
| College Education (4-year degree or more) | 1.2259 |
| Age minus 25 and Female | -0.0041 |
| Age minus 25 and High School | -0.0202 |
| Age minus 25 and College | -0.0206 |
| Female and High School | -0.4233 |
| Female and College | -0.6505 |
| Estimated from combined 1996-1997 BRFSS data. The reference category for educational attainment is no high school diploma. | |

**Quit-types and smoking cessation medications**

The cessation probability that is finally applied for each agent also accounts for the relative effectiveness of the agent’s quit strategy using a three-step process. First, one of six quit strategies is assigned to each person according to probabilities of self-reported methods used to quit smoking by insurance in the NHIS as shown in Table S1.4.[^10^](#_ENREF_10) Quit strategies indirectly reflect demographic characteristics through the assignment of insurance status. Second, the unassisted quit rate for all persons of similar age, sex, and education level is determined using Bayes’ Rule. Finally, the agent’s actual cessation rate is determined by scaling the estimated unassisted quit rate by the relative rate of agent’s assigned quit type. The relative quit rates of brief medical counseling,[^11^](#_ENREF_11)^,^[^12^](#_ENREF_12) Rx NRT[^13^](#_ENREF_13), bupropion[^13^](#_ENREF_13), and varenicline^[13](#_ENREF_13)^ are 1.32, 1.60, 1.69, and 2.27. That of OTC NRT is equal to Rx NRT based on mixed evidence on equivalence of effectiveness.[^13-18^](#_ENREF_13)

| **Table S1.4. Quit types (quit strategies) by insurance type** | | | | |  |  |
| --- | --- | --- | --- | --- | --- | --- |
|  | **Completely unassisted** | **Brief counseling alone** | **OTC NRT (gum or patch)** | **Prescription NRT (spray or inhaler) with brief counseling** | **Bupropion (Zyban) with brief counseling** | **Varenicline (Chantix) with brief counseling** |
| Medicaid | 46.0% | 26.0% | 16.9% | 0.7% | 2.3% | 8.1% |
| Medicare | 41.0% | 29.0% | 18.1% | 0.8% | 2.5% | 8.6% |
| Private | 49.0% | 15.0% | 21.4% | 0.9% | 2.9% | 10.2% |
| Other | 48.0% | 17.0% | 21.4% | 1.0% | 3.0% | 10.0% |
| Uninsured | 65.0% | 15.0% | 19.9% | 0.0% | 0.0% | 0.0% |

Cessation probabilities are not modeled as a conditional probability of a quit attempt. Rather they are estimated as described above, and quit attempt probabilities are derived by age, sex, and race-ethnicity from the NHIS. To determine costs, failed attempts are defined as the difference between quit attempts and cessation.

### Relapse rates

Relapse after quitting tobacco use is time-sensitive. The longer a person has successfully quit smoking, the less likely they are to relapse. We constructed the relapse curve represented by the conditional relapse probabilities shown in Figure S1.4 based on reporting in five retrospective and prospective studies and reviews.[^19-23^](#_ENREF_19) We fit a log-linear relapse curve to these estimates as shown in Figure S1.4 for use in the model. These relapse rates are applied to all quits in the model, whether they are part of the baseline model or are induced by a clinical intervention, program or policy change.

In using relapse estimates from the literature, it was important to recognize that the probability of cessation we estimated from BRFSS reflects smokers who quit anytime in the year prior to the survey and remained non-smoking at the time of the survey. Therefore, these cessation probabilities already reflect some initial relapse. The estimates reflect a range of former smokers who quit from between one week and 51 weeks prior to the survey. Therefore, in applying relapse rates from the literature, we sought an estimate for the first year of relapse that reflected the probability of relapse conditional on having not relapsed for an average of six months.

**Figure S1.4. Relapse curve: the probability of relapse among previous
years’ former smokers**


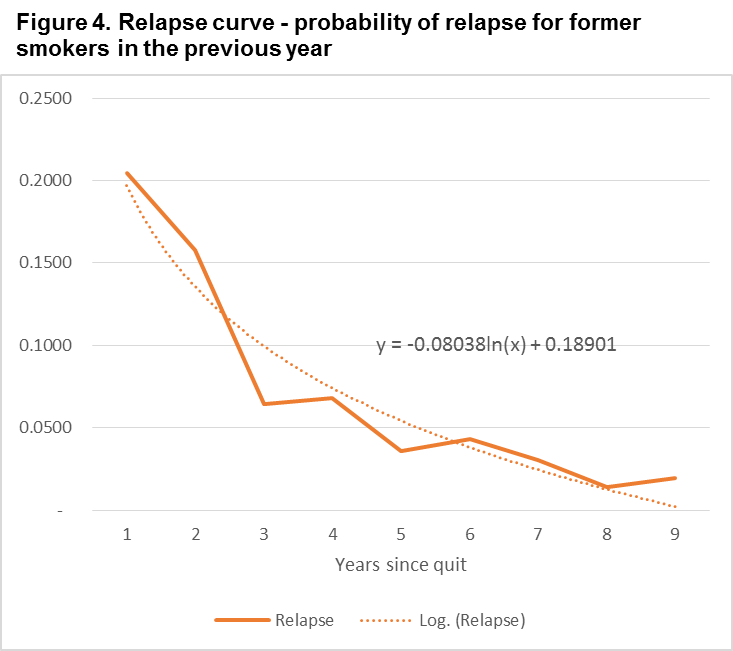


# The Health Impact Module

The Health Impact Module determines how the smoking behavior of simulated individuals affects disease incidence, morbidity and mortality. In assessing policy or program impact, we compare the disease outcomes of each agent that occur in the baseline scenario (without the policy or program) to those that occur in the policy or program scenario. Population-wide estimates of an intervention’s impact are determined by aggregating individual effects.

The Health Impact Module tracks outcomes across a variety of tobacco-related diseases simultaneously using age-, sex-, and smoking-status-based risks derived from Smoking-Attributable Mortality, Morbidity, and Economic Costs (SAMMEC) as reported in the 2014 Surgeon General’s report on tobacco.[^24^](#_ENREF_24) This approach provides a broad accounting of smoking attributable risks and diseases.

### SAMMEC-based disease and burden estimation

Our approach to attributing events by age, sex and smoking status has been described elsewhere in the context of creating alternative estimates of smoking-attributable medical costs by age, sex and smoking status.[^25^](#_ENREF_25) The mathematics used to implement the approach described below are available in the appendix of that article.

### Smoking-attributable mortality risk by age, sex and smoking status

We obtained the age-specific (five-year age groups from age 35 to 84, and 85+) and sex-specific mortality risks of smoking-attributable conditions for Minnesota from compressed mortality files for 1996-1998.[^26^](#_ENREF_26) Smoking-attributable conditions are the 12 cancers, four cardiovascular disease categories, diabetes, and four respiratory disease categories identified in Smoking-Attributable Mortality, Morbidity, and Economic Costs (SAMMEC)[^24^](#_ENREF_24) as shown in Table S1.5. To distribute mortality risk by age, sex and smoking status, we applied age and sex-specific smoking-attributable relative risks for each disease category that we also obtained from SAMMEC. Relative risks are assumed to equal 1.0 for ages below 35 in SAMMEC, and hence there is no smoking-attributable disease prior to age 35 in the model. The risks are available from the authors by request, in an Excel spreadsheet.

### Smoking-attributable disease risk by age, sex and smoking status

State-specific non-fatal disease events are not readily available by sex and detailed age group. Therefore, to estimate disease events by smoking status, we first assessed the number and distribution of smoking-attributable disease events in the U.S. population by age and sex. First, smoking-related disease events were obtained by sex and five-year age groups. We obtained the incidence of cancers from the Surveillance, Epidemiology, and End Results (SEER) Program of the National Cancer Institute[^27^](#_ENREF_27) , and hospitalizations for cardiovascular disease, diabetes and respiratory disease from the National Hospital Discharge Survey (NHDS).[^28^](#_ENREF_28) Hospitalizations were selected if their first-listed discharge diagnosis was for a smoking-attributable disease as defined in SAMMEC.[^24^](#_ENREF_24) We used these U.S. data in ModelHealth: Tobacco to obtain U.S. case-fatality rates by age group. We then multiplied fatality rates of smoking-attributable conditions for Minnesota by the inverse of these case-fatality rates to approximate case rates for Minnesota. The case-fatality rates are available by request from the authors in an Excel spreadsheet.

As with mortality data, neither SEER cancer data nor the NHDS contain cigarette smoking status that could be used to directly calculate disease events by smoking status. Therefore, relative risks by smoking status are needed to apportion rates by smoking status. Relative risks of non-fatal events are not available for a broad range of diseases from a standardized source. Therefore, the mortality relative risks provided in SAMMEC were used to distribute the age- and sex-specific disease events among never, current and former smokers. The use of mortality relative risks as a proxy for nonfatal event relative risks implicitly assumes that the event-fatality rate is constant across smoking status groups. If this is not the case, then our calculations may over-state or under-state the benefits of quitting. The risks are available by request from the authors in an Excel spreadsheet.

### Smoking-attributable diseases, health utilities, and duration

The model tabulated quality adjusted life years (QALYs) for each smoking-attributable event by assigning a disease duration and associated decrement in quality of life. Given incidence of a particular disease, severity, and final outcome (death or recovery), episode duration is determined. Disease-specific quality of life (QoL) decrements are imposed during disease episodes to capture morbidity. A maximum decrement of 0.5 is applied to individuals with more than one simultaneously occurring condition.

| **Table S1.5: Summary of diseases included in ModelHealth: Tobacco MN** | | | | |
| --- | --- | --- | --- | --- |
|  | **Episode Duration^a^** | | **Quality Adjusted Life Year Decrements** | |
|  | **Fatal** | **Non-fatal** | **Initial Year of Event^b^** | **Subsequent Years** |
| **CANCERS** |  |  |  |  |
| Lip, Oral Cavity, Pharynx | 2 | 5 | 0.2 | 0.2 |
| Esophagus | 1 | 5 | 0.3 | 0.3 |
| Stomach | 1 | 5 | 0.3 | 0.3 |
| Colorectal Cancer | 2 | 5 | 0.2 | 0.2 |
| Liver | 1 | 5 | 0.3 | 0.3 |
| Pancreas | 1.24 | 5 | 0.3 | 0.3 |
| Larynx | 2 | 5 | 0.3 | 0.3 |
| Trachea_Lung_Bronchus | 2 | 5 | 0.3 | 0.3 |
| Cervix Uteri | 4 | 5 | 0.2 | 0.2 |
| Kidney and Renal Pelvis | 4.7 | 5 | 0.2 | 0.2 |
| Urinary Bladder | 4.7 | 5 | 0.2 | 0.2 |
| Acute Myeloid Leukemia | 4.6 | 5 | 0.2 | 0.2 |
| **CVD** |  |  |  |  |
| Ischemic Heart Disease | 0 | 0.5 | 0.1500 |  |
| Other Heart Disease | 5 | 0.0769 | 0.0231 |  |
| Cerebrovascular Disease Stroke^c^ | 1 | until death | 0.4000 | 0.4 |
| Other Cardiovascular Disease | 5 | 0.0769 | 0.0231 | 0.3 |
| Diabetes | 5 | until death | 0.1 |  |
| **Respiratory Disease** |  |  |  | 0.1 |
| Pneumonia, Influenza, TB | 0 | 0.0384 | 0.0115 |  |
| Bronchitis Emphysema^c^ | 5 | until death | 0.2 | 0.2 |
| ^a^Durations are rounded up to the nearest cycle. Episodes with 0 duration indicate instant death and no decrement applied. | | | | |
| ^b^For CVD and Respiratory Diseases, the initial year decrement is scaled to reflect partial year episode | | | | |
| ^c^Following initial episode, agent remains at risk for death in future cycles. | | | | |

Table S1.5 lists the diseases included in the health impact module, with their assumed duration and quality of life decrement. The duration of fatal cancer episodes ranges from 1 to 5 years with applicable decrements applied during the fatal episode. The duration of a non-fatal cancer episode was assumed to be five years across all cancers. Quality of Life decrements were the same for both fatal and non-fatal cancer episodes and ranged from 0.2 to 0.3 QALYs based on the standardized health utilities for chronic and acute conditions used in analyses for the National Commission on Prevention Priorities.[^29^](#_ENREF_29) Once a non-fatal cancer episode ends, the individual is at risk of another episode of that cancer with the same probability of remission if a new case does develop.

Cardiovascular and respiratory disease are modeled as both fatal events and chronic episodes with quality of life decrements ranging from .01 (influenza) to .4 (stroke). Events resulting in death have a duration of one year with a half-cycle correction, which has the effect of reducing the average duration to six months. The corresponding quality of life decrement for chronic cardiovascular and respiratory diseases is imposed every year following the event. Individuals experiencing a non-fatal cardiovascular and/or respiratory event could experience a repeat event. Their risk for a repeat event is the same as that of experiencing the initial event. For example, a nonfatal cerebrovascular disease episode (i.e. stroke) results in a quality of life decrement of 0.4 QALYs every cycle following that event. The individual experiencing that initial stroke remains at risk of another stroke in subsequent years with a risk of the next stroke being fatal.

### Competing causes of death

During each cycle, individuals are also subject to age-specific probabilities of death from other causes. These probabilities are approximated by subtracting the combined probabilities of death from smoking-attributable conditions obtained from compressed mortality data[^30^](#_ENREF_30) from overall mortality rates by age obtained from U.S. life tables.[^31^](#_ENREF_31)

#### Recent quitters and lagged change in disease risk

Recent quitters have smoking-attributable health risks within 25% of that of current smokers for approximately four years after quitting although the delay for cardiovascular disease benefits may be less.[^32^](#_ENREF_32) Therefore, ModelHealth: Tobacco MN imposes a four-year lag between when a smoker quits and when a smoker’s risks for cancers and respiratory disease are reduced from those of current smokers to those of former smokers.

### Costs and productivity

ModelHealth: Tobacco MN tracks both direct medical care expenditures and indirect productivity effects of smoking, though productivity effects are not necessarily used in all analyses. Because state-specific estimates are not available by age, sex and insurance status, we scaled U.S. estimates to Minnesota as described below.

### Smoking-attributable medical costs

The model includes only the costs of smoking-attributable medical care. It does not assign costs to never smokers, and the costs of current and former smokers included in the model are net of average costs for like individuals who are never smokers. For the United States as a whole, we estimated the medical costs of smoking from observed associations between smoking status and medical costs in the Medical Expenditure Panel Survey (MEPS), using smoking status from linked NHIS responses.[^33^](#_ENREF_33) We followed the method of Levy et al.,[^34^](#_ENREF_34) including controlling for potentially confounding factors in a two-part model using a gamma distribution and a log-link in the second part. However, we combined multiple years of data (2001-2010) to create more stable estimates for age, sex and smoking status subgroups; we also estimated separate models by primary insurer to determine smoking costs by the primary insurer type. MEPS and other claims data are complicated by higher utilization of some former smokers whose quits were prompted by diagnoses that lead to increased healthcare utilization in the years following their successful quits. For former smokers, we fit an exponential function to the relationship of current and former risk based on time since quit, as reported by the Congressional Budget Office (Figure 3-5 in CBO report). We applied this function to the costs for current smokers that we estimated from MEPS data to obtain estimates of what the medical costs of former smokers would be by age, sex and time since quit if they had a proactive quit:

y = 0.9927 - 1.086e(-0.1171t),

where y is the portion of a current smokers’ smoking-attributable costs that is reduced according to years since quit (=t). Thus each former’s smoker cost is calculated as a portion of current smokers’ costs with the same age, sex and insurance status as estimated from MEPS. The function implies that 50% of the excess medical costs of smoking are eliminated in the 7^th^ year after quit (the functions’ “half-life”), and 90% is eliminated by the 21^st^ year.

The CBO ‘index’ was constructed based on a literature review of the relationship between time since quit and reduction in mortality risk for smoking-attributable diseases, weighted by each disease’s share of smoking-attributable mortality. In their analysis, the CBO applied its index to both mortality risk and medical care expenditures due to lack of better information on the expenditure trend of healthy quitters. Therefore, smoking-attributable medical costs of former smokers based on the function above must be recognized as an approximation.

Per-person smoking attributable expenditures do not change over time other than with changes in age and smoking status. We inflated U.S. estimates of the costs of current smoking to 2017 U.S. dollars and scaled the estimates using the ratio of Minnesota to U.S. per capita healthcare expenditures.[^35^](#_ENREF_35) Table S1.6 provides the resulting smoking-attributable costs for current smokers by age and sex for the United States. In the model, former smoker costs will vary be age, sex and year since quit per the equation specified above. For illustrative purposes, Table A.4 also provides costs of former smokers who have been quit for five years.

| **Table S1.6. Smoking-attributable medical costs by age, gender, smoking status for MN ($2017)** | | | | |
| --- | --- | --- | --- | --- |
| **Age categories (in years)** | **Current smoker** | | **Former smoker, fifth year^a^** | |
|  | **Male** | **Female** | **Male** | **Female** |
| **Private insurance** | | | | |
| 0-34 | 0 | 0 | 0 | 0 |
| 35-44 | 1,092 | 1,338 | 668 | 819 |
| 45-54 | 1,399 | 1,658 | 856 | 1,014 |
| 55-64 | 1,766 | 2,039 | 1,081 | 1,248 |
| 65-74 | 2,206 | 2,492 | 1,349 | 1,525 |
| 75-84 | 2,727 | 3,034 | 1,669 | 1,857 |
| 85+ | 3,024 | 3,345 | 1,851 | 2,047 |
| **Medicare insurance** | | | | |
| 0-34 | 0 | 0 | 0 | 0 |
| 35-44 | 1,281 | 1,508 | 784 | 923 |
| 45-54 | 1,614 | 1,851 | 988 | 1,133 |
| 55-64 | 2,009 | 2,261 | 1,229 | 1,384 |
| 65-74 | 2,480 | 2,753 | 1,518 | 1,684 |
| 75-84 | 3,042 | 3,340 | 1,861 | 2,044 |
| 85+ | 3,362 | 3,676 | 2,058 | 2,249 |
| **Medicaid insurance** | | | | |
| 0-34 | 0 | 0 | 0 | 0 |
| 35-44 | 3,004 | 3,488 | 1,837 | 2,135 |
| 45-54 | 3,761 | 4,272 | 2,302 | 2,615 |
| 55-64 | 4,663 | 5,210 | 2,854 | 3,188 |
| 65-74 | 5,734 | 6,330 | 3,509 | 3,873 |
| 75-84 | 7,015 | 7,671 | 4,294 | 4,696 |
| 85+ | 7,747 | 8,441 | 4,742 | 5,167 |
| **Uninsured** | | | | |
| 0-34 | 0 | 0 | 0 | 0 |
| 35-44 | 464 | 680 | 284 | 416 |
| 45-54 | 642 | 881 | 392 | 540 |
| 55-64 | 863 | 1,125 | 529 | 688 |
| 65-74 | 1,134 | 1,413 | 694 | 865 |
| 75-84 | 1,465 | 1,756 | 896 | 1,075 |
| 85+ | 1,653 | 1,950 | 1,012 | 1,194 |
| **Other/Multiple insurance** | | | | |
| 0-34 | 0 | 0 | 0 | 0 |
| 35-44 | 1,907 | 2,213 | 1,167 | 1,354 |
| 45-54 | 2,386 | 2,711 | 1,461 | 1,660 |
| 55-64 | 2,959 | 3,307 | 1,811 | 2,023 |
| 65-74 | 3,639 | 4,017 | 2,228 | 2,458 |
| 75-84 | 4,452 | 4,868 | 2,725 | 2,979 |
| 85+ | 4,917 | 5,356 | 3,009 | 3,278 |
| ^a^Costs of former smokers are determined by time since quit as described in the text. Former smoker costs here are illustrated by those with five years since their quit. | | | | |

## Productivity

The simulation model incorporated three sources of productivity: premature mortality; absenteeism, or days of lost productivity not associated with exit from labor force; and presenteeism, or being at less-than-full working capacity during days of work. Each of these categories can have two dimensions: lost labor force productivity and lost non-labor force productivity. Non-labor force productivity could be further divided into time spent producing goods and services outside the formal labor market, and time spent in leisure activity. However, productivity losses in the model are limited to lost labor force productivity and time spent producing services outside of the labor force.

The model assigns a positive productivity for each year of adult life that varies by age but not sex or smoking status as shown in Table S1.7. Current and former smokers are assigned a decrement to this productivity to account for absenteeism and presenteeism as described below.

We implemented an approach to productivity that combines the highest-quality literature sources available to estimate potential productivity losses from smoking. Simulated individuals may experience premature death from smoking-attributable disease. The difference between age of death with and without intervention determines the number of years of premature mortality. We valued the productivity of each year of life using estimates by age group (not differentiated by sex) reported by Grosse et al.[^37^](#_ENREF_37) updated through 2012 for changes in national average of employee earnings and benefits.[^38^](#_ENREF_38) Per-person productivity estimates do not change over time, other than with changes in age and smoking status. For ModelHealth: Tobacco MN, we inflation adjusted these estimates to 2017 U.S. dollars and scaled the U.S. measures of productivity by the ratio of Minnesota to U.S. per capita earnings,[^39^](#_ENREF_39) These productivity estimates for the Minnesota are summarized in Table S1.7.

| **Table A.5. Annual Productivity of MN Adults ($2017)** | | | | |
| --- | --- | --- | --- | --- |
| **Age group** | **Per-person annual market  compensation** | **Per-person annual household  production value** | **Per-person total annual  production value** |  |
| 35–39 | 61,956 | 22,328 | 84,284 |  |
| 40–44 | 64,373 | 21,152 | 85,524 |  |
| 45–49 | 64,889 | 19,369 | 84,259 |  |
| 50–54 | 63,913 | 17,621 | 81,534 |  |
| 55–59 | 52,410 | 18,773 | 71,183 |  |
| 60–64 | 37,779 | 19,647 | 57,426 |  |
| 65–69 | 13,545 | 20,911 | 34,455 |  |
| 70–74 | 7,196 | 20,632 | 27,827 |  |
| 75–79 | 3,742 | 19,586 | 23,329 |  |
| 80+ | 2,096 | 15,535 | 17,631 |  |
| Source: [^37^](#_ENREF_37)^,^[^38^](#_ENREF_38). |  |  |  |  |

The estimates of Grosse et al.[^37^](#_ENREF_37) include household productivity reported separately from workplace productivity, as measured by market compensation that includes employee pay and benefits. Both household and market productivity estimates are included in ModelHealth: Tobacco MN. These estimates reflect the average of those in and out of the labor force. We therefore we apply them to all individuals in the models, regardless of employment status.

Few estimates of absenteeism and presenteeism are available across multiple conditions in a generalizable population. Mitchell and Bates[^40^](#_ENREF_40) estimated combined absenteeism and presenteeism costs in one million employees for 13 conditions and four risk factors, based on Work Limitations Questionnaire (WLQ), but they did not report those costs separately. Mitchell and Bates[^40^](#_ENREF_40) adjusted salary and benefit valuation up by a factor of 1.6 to reflect the “multiplier” impact of absenteeism and presenteeism on work team performance as estimated by Nicholson et al.[^41^](#_ENREF_41) This multiplier is still reflected in our adjusted estimates. A more recent analysis suggests that compensating efforts by the ill employee in off-work hours and by coworkers may more than offset the negative impact of a team member on productivity of the rest of the work team.[^42^](#_ENREF_42)

Several adjustments were needed to apply these estimates of absenteeism and presenteeism costs to the model. Mitchell and Bates[^40^](#_ENREF_40) reported average days lost across all age groups (ages 18-70). In ModelHealth: Tobacco MN, all smoking-attributable disease occurs after age 35. To improve internal consistency between disease occurrence, disease costs and productivity costs, we assign zero absenteeism and presenteeism costs to ages 15-34, and we reapportion all absenteeism and presenteeism days to the 35+ age group. Another difference is that Mitchell and Bates[^40^](#_ENREF_40) estimated the average days *per employee*; in comparison, Grosse et al.[^37^](#_ENREF_37) reported average market productivity across all adults employed and not employed. To implement these estimates in the same manner in the model, we adjusted Mitchell and Bates’ estimates downward by multiplying them by the portion of the U.S. population ages 25 to 64 who are employed. This allows us to apply the estimates of absenteeism and presenteeism to all individuals in the model, regardless of employment status, without overstating population effects. This in analogous to how population average market and household productivity estimates from Grosse et al.[^37^](#_ENREF_37) are applied to all individuals, regardless of labor market status, as described above. As a result, population-wide effects from the model are accurate, but the model does not have the ability to accurately report productivity measures stratified by labor status. We also adjusted estimates to 2012 dollars and added productivity growth over time in the same manner described above for productivity losses associated with premature mortality. For ModelHealth: Tobacco MN, we scaled the estimates by the ratio of Minnesota to U.S. per capita earnings.[^39^](#_ENREF_39) The result is $426 per year in combined absenteeism and presenteeism per year for each current smoker. Absenteeism and presenteeism productivity losses for former smokers are calculated as a portion of those of current smokers, varying as a function of time since quit as described for smoking-attributable medical expenditures above.

## References

1. Maciosek MV, LaFrance AB, Dehmer SP, et al. Health Benefits and Cost-Effectiveness of Brief Clinician Tobacco Counseling for Youth and Adults. *Ann Fam Med.* 2017;15(1):37-47.

2. Maciosek MV, LaFrance AB, Dehmer SP, et al. Updated Priorities Among Effective Clinical Preventive Services. *Annals of Family Medicine.* 2017;15(1):12-22.

3. US Census Bureau. Current Population Survey, Supplemental Surveys. 2018; <https://www.census.gov/programs-surveys/cps/about/supplemental-surveys.html>. Accessed Sep 18, 2018.

4. King M, Ruggles S, Alexander JT, et al. Integrated Public Use Microdata Series, Current Population Survey: 2009-2012. Minneapolis, MN: Minnesota Population Center2014.

5. Survey of Income and Program Participation. 2008. <http://www.census.gov/programs-surveys/sipp/>. Accessed 2014.

6. Minnesota Center for Health Statistics, Minnesotat Department of Health. Minnesota Youth Tobacco Survey. <http://www.health.state.mn.us/divs/chs/surveys/tobacco/index.html>.

7. Nelson DE, Mowery P, Asman K, et al. Long-term trends in adolescent and young adult smoking in the United States: metapatterns and implications. *Am J Public Health.* 2008;98(5):905-915.

8. Centers for Disease Control and Prevention. Behavioral Risk Factor Surveillance System. <http://www.cdc.gov/brfss/>. Accessed November 13, 2017.

9. *Tobacco Use in Minnesota: 2014 Update.* Minneapolis, MN: ClearWay Minnesota and Minnesota Department of Health; January 2015.

10. National Center for Health Statistics. National Health Interview Survey. Hyattsville, Maryland: National Center for Health Statistics, Centers for Disease Control and Prevention2017.

11. Meyer C, Ulbricht S, Baumeister SE, et al. Proactive interventions for smoking cessation in general medical practice: a quasi-randomized controlled trial to examine the efficacy of computer-tailored letters and physician-delivered brief advice. *Addiction.* 2008;103(2):294-304.

12. Solberg LI, Maciosek MV, Edwards NM, Khanchandani HS, Goodman MJ. Repeated tobacco-use screening and intervention in clinical practice health impact and cost effectiveness. *Am J Prev Med.* 2006;31(1):62-71.

13. Cahill K, Stevens S, Perera R, Lancaster T. Pharmacological interventions for smoking cessation: an overview and network meta-analysis. *Cochrane Database Syst Rev.* 2013;5:CD009329.

14. Shiffman S, Rolf CN, Hellebusch SJ, et al. Real-world efficacy of prescription and over-the-counter nicotine replacement therapy. *Addiction.* 2002;97(5):505-516.

15. Hughes JR, Shiffman S, Callas P, Zhang J. A meta-analysis of the efficacy of over-the-counter nicotine replacement. *Tob Control.* 2003;12(1):21-27.

16. Amodei N, Lamb RJ. Over-the-counter nicotine replacement therapy: can its impact on smoking cessation be enhanced? *Psychol Addict Behav.* 2008;22(4):472-485.

17. Schnoll RA, Rukstalis M, Wileyto EP, Shields AE. Smoking cessation treatment by primary care physicians: An update and call for training. *Am J Prev Med.* 2006;31(3):233-239.

18. Shiffman S, Sweeney CT, Rohay JM, Ertischek MD. Physician involvement in recommending over-the-counter nicotine replacement therapy. *Am J Prev Med.* 2007;32(4):358-359.

19. *The Health Benefits of Smoking Cessation.* Rockville, MD: U.S. Department of Health and Human Services Public Health Service Centers for Disease Control Center for Chronic Disease Prevention and Health Promotion Office on Smoking and Health; 1990; No. DHHS Publication No. (CDC) 90-8416.

20. Wetter DW, Cofta-Gunn L, Fouladi RT, Cinciripini PM, Sui D, Gritz ER. Late relapse/sustained abstinence among former smokers: a longitudinal study. *Prev Med.* 2004;39(6):1156-1163.

21. Herd N, Borland R, Hyland A. Predictors of smoking relapse by duration of abstinence: findings from the International Tobacco Control (ITC) Four Country Survey. *Addiction.* 2009;104(12):2088-2099.

22. Hughes JR, Peters EN, Naud S. Relapse to smoking after 1 year of abstinence: a meta-analysis. *Addict Behav.* 2008;33(12):1516-1520.

23. Gilpin EA, Pierce JP, Farkas AJ. Duration of smoking abstinence and success in quitting. *J Natl Cancer Inst.* 1997;89(8):572-576.

24. U.S. Department of Health and Human Services. The Health Consequences of Smoking—50 Years of Progress: A Report of the Surgeon General, 2014. Atlanta, GA: U.S. Department of Health and Human Services, Centers for Disease Control and Prevention, National Center for Chronic Disease Prevention and Health Promotion, Office on Smoking and Health; 2014.

25. Maciosek MV, Xu X, Butani AL, Pechacek TF. Smoking-attributable medical expenditures by age, sex, and smoking status estimated using a relative risk approach. *Prev Med.* 2015;77:162-167.

26. CDC Wonder - Compressed Mortality File - Underlying cause-of-death. <http://wonder.cdc.gov/cmf-icd10.html>. Accessed [2011-05-02.

27. Horner MJ RL, Krapcho M, Neyman N, Aminou R, Howlader N, Altekruse SF, Feuer EJ, Huang L, Mariotto A, Miller BA, Lewis DR, Eisner MP, Stinchcomb DG, Edwards BK (eds). SEER Cancer Statistics Review, 1975-2006. 2009; <http://seer.cancer.gov/csr/1975_2006/>. Accessed April, 12 2011.

28. DeFrances CJ, Hall MJ. 2002 National Hospital Discharge Survey. *Adv Data.* 2004(342):1-29.

29. Maciosek MV, Coffield AB, McGinnis JM, et al. Methods for priority setting among clinical preventive services. *Am J Prev Med.* 2001;21(1):10-19.

30. Centers for Disease Control and Prevention. Compressed Mortality File 1999-2006. CDC WONDER On-line Database, compiled from Compressed Mortality File 1999-2006 Series 20 No. 2L, 2009. <http://wonder.cdc.gov/cmf-icd10.html>. Accessed Dec 8, 2009 12:52:14 PM.

31. Arias E. *United States Life Tables, 2010.* Centers for Disease Control and Prevention, Division of Vital Statistics; November 6, 2104 2014.

32. Congressional Budget Office. Raising the Excise Tax on Cigarettes: Effects on Health and the Federal Budget. Washington: Congressional Budget Office; 2012.

33. Chowdhury S MS, Wun L. *Linking Medical Expenditure Panel Survey to the National Health Interview Survey: Weighting and Estimation. Working Paper No. 12005.* Rockville, MD: Agency for Healthcare Research and Quality;2012.

34. Levy DE NJ. Assessing the effects of tobacco policy changes on smoking-related health expenditures. In: Bearman PS NK, Wright L, ed. *After Tobacco: What Would Happen If Americans Stopped Smoking?* New York: Columbia University Press; 2011:256–289.

35. Health Expenditures by State of Residence. Centers for Medicare & Medicaid Services; 2011. <http://www.cms.gov/NationalHealthExpendData/downloads/resident-state-estimates.zip>.

36. Congressional Budget Office. *Raising the Excise Tax on Ciagrettes: Effects on Health and the Federal Budget. Congress of the United States, Congressional Budget Office.*  June 2012.

37. Grosse SD, Krueger KV, Mvundura M. Economic productivity by age and sex: 2007 estimates for the United States. *Med Care.* 2009;47(7 Suppl 1):S94-103.

38. Bureau of Labor Statistics. Employment Cost Index for the Wages and Salaries of all Civilian Workers. Bureau of Labor Statistics. <ftp://ftp.bls.gov/pub/suppl/eci.echistrynaics.txt>. Accessed October 30, 2013.

39. Quarterly Census of Employment & Wages. <https://www.bls.gov/data/>. Accessed 10/13/2017.

40. Mitchell RJ, Bates P. Measuring health-related productivity loss. *Popul Health Manag.* 2011;14(2):93-98.

41. Nicholson S, Pauly MV, Polsky D, Sharda C, Szrek H, Berger ML. Measuring the effects of work loss on productivity with team production. *Health Econ.* 2006;15(2):111-123.

42. Krol M, Brouwer WB, Severens JL, Kaper J, Evers SM. Productivity cost calculations in health economic evaluations: correcting for compensation mechanisms and multiplier effects. *Soc Sci Med.* 2012;75(11):1981-1988.

# Supplemental Tables

## Coefficients estimated for the health insurance submodel

## Initial smoking status at time 0 (at model initiation)

| **Initial probabilities of current smoking** | | | | | | | | |
| --- | --- | --- | --- | --- | --- | --- | --- | --- |
|  | **Male** | | | | **Female** | | | |
| **Age** | **White, non-Hispanic** | **Black, non-Hispanic** | **Hispanic** | **Other*** | **White, non-Hispanic** | **Black, non-Hispanic** | **Hispanic** | **Other*** |
| 9 | 0.0033 | 0.0023 | 0.0021 | 0.0019 | 0.0023 | 0.0014 | 0.0009 | 0.0013 |
| 10 | 0.0077 | 0.0053 | 0.0048 | 0.0044 | 0.0053 | 0.0033 | 0.0020 | 0.0031 |
| 11 | 0.0151 | 0.0104 | 0.0094 | 0.0086 | 0.0104 | 0.0065 | 0.0039 | 0.0061 |
| 12 | 0.0228 | 0.0157 | 0.0142 | 0.0129 | 0.0157 | 0.0098 | 0.0058 | 0.0092 |
| 13 | 0.0299 | 0.0205 | 0.0186 | 0.0170 | 0.0206 | 0.0129 | 0.0076 | 0.0120 |
| 14 | 0.0524 | 0.0349 | 0.0316 | 0.0288 | 0.0552 | 0.0346 | 0.0205 | 0.0322 |
| 15 | 0.0774 | 0.0532 | 0.0481 | 0.0439 | 0.0718 | 0.0450 | 0.0267 | 0.0419 |
| 16 | 0.1188 | 0.0816 | 0.0738 | 0.0673 | 0.1131 | 0.0709 | 0.0421 | 0.0661 |
| 17 | 0.1664 | 0.1142 | 0.1033 | 0.0943 | 0.1621 | 0.1016 | 0.0603 | 0.0947 |
| 18 | 0.1606 | 0.1103 | 0.0997 | 0.0910 | 0.1624 | 0.1018 | 0.0604 | 0.0949 |
| 19 | 0.2015 | 0.1476 | 0.1247 | 0.1185 | 0.2148 | 0.1360 | 0.0793 | 0.1217 |
| 20 | 0.2158 | 0.1590 | 0.1363 | 0.1255 | 0.2332 | 0.1436 | 0.0847 | 0.1257 |
| 21 | 0.2294 | 0.1692 | 0.1450 | 0.1391 | 0.2497 | 0.1573 | 0.0892 | 0.1298 |
| 22 | 0.2432 | 0.1812 | 0.1522 | 0.1589 | 0.2648 | 0.1688 | 0.0956 | 0.1347 |
| 23 | 0.2561 | 0.1944 | 0.1641 | 0.1668 | 0.2809 | 0.1806 | 0.1008 | 0.1387 |
| 24 | 0.2721 | 0.2054 | 0.1676 | 0.1797 | 0.2980 | 0.1921 | 0.1050 | 0.1425 |
| 25 | 0.2845 | 0.2220 | 0.1741 | 0.1925 | 0.3125 | 0.2048 | 0.1107 | 0.1459 |
| 26 | 0.2934 | 0.2277 | 0.1815 | 0.2053 | 0.3261 | 0.2143 | 0.1095 | 0.1354 |
| 27 | 0.2928 | 0.2327 | 0.1873 | 0.2174 | 0.3279 | 0.2215 | 0.1128 | 0.1293 |
| 28 | 0.2960 | 0.2360 | 0.1878 | 0.2185 | 0.3309 | 0.2180 | 0.1201 | 0.1241 |
| 29 | 0.2993 | 0.2398 | 0.1822 | 0.2163 | 0.3321 | 0.2232 | 0.1222 | 0.1133 |
| 30 | 0.3005 | 0.2407 | 0.1834 | 0.2054 | 0.3330 | 0.2208 | 0.1248 | 0.1128 |
| 31 | 0.2995 | 0.2464 | 0.1838 | 0.1994 | 0.3307 | 0.2247 | 0.1250 | 0.1087 |
| 32 | 0.2949 | 0.2493 | 0.1908 | 0.1982 | 0.3297 | 0.2238 | 0.1187 | 0.1087 |
| 33 | 0.2920 | 0.2450 | 0.1924 | 0.2038 | 0.3270 | 0.2298 | 0.1137 | 0.0991 |
| 34 | 0.2887 | 0.2403 | 0.1951 | 0.2062 | 0.3225 | 0.2310 | 0.1169 | 0.0957 |
| 35 | 0.2866 | 0.2377 | 0.1888 | 0.2121 | 0.3179 | 0.2354 | 0.1181 | 0.0925 |
| 36 | 0.2830 | 0.2365 | 0.1890 | 0.2113 | 0.3124 | 0.2347 | 0.1189 | 0.0975 |
| 37 | 0.2797 | 0.2358 | 0.1870 | 0.2000 | 0.3088 | 0.2334 | 0.1175 | 0.0932 |
| 38 | 0.2768 | 0.2352 | 0.1845 | 0.1956 | 0.3046 | 0.2307 | 0.1172 | 0.0887 |
| 39 | 0.2709 | 0.2388 | 0.1877 | 0.1933 | 0.2984 | 0.2314 | 0.1192 | 0.0870 |
| 40 | 0.2682 | 0.2348 | 0.1888 | 0.1840 | 0.2923 | 0.2347 | 0.1203 | 0.0944 |
| 41 | 0.2629 | 0.2381 | 0.1905 | 0.1853 | 0.2850 | 0.2380 | 0.1283 | 0.0911 |
| 42 | 0.2597 | 0.2397 | 0.1858 | 0.1893 | 0.2787 | 0.2402 | 0.1299 | 0.0997 |
| 43 | 0.2547 | 0.2399 | 0.1835 | 0.1965 | 0.2730 | 0.2458 | 0.1297 | 0.0945 |
| 44 | 0.2499 | 0.2426 | 0.1861 | 0.1957 | 0.2686 | 0.2495 | 0.1284 | 0.0902 |
| 45 | 0.2462 | 0.2412 | 0.1897 | 0.1918 | 0.2638 | 0.2450 | 0.1290 | 0.1031 |
| 46 | 0.2373 | 0.2359 | 0.1898 | 0.1866 | 0.2590 | 0.2416 | 0.1311 | 0.1061 |
| 47 | 0.2316 | 0.2418 | 0.1787 | 0.1860 | 0.2541 | 0.2384 | 0.1309 | 0.1040 |
| 48 | 0.2259 | 0.2462 | 0.1751 | 0.1837 | 0.2484 | 0.2386 | 0.1377 | 0.1060 |
| 49 | 0.2221 | 0.2461 | 0.1669 | 0.1812 | 0.2457 | 0.2331 | 0.1369 | 0.1036 |
| 50 | 0.2173 | 0.2508 | 0.1649 | 0.1730 | 0.2401 | 0.2382 | 0.1387 | 0.1093 |
| 51 | 0.2140 | 0.2559 | 0.1591 | 0.1653 | 0.2374 | 0.2348 | 0.1352 | 0.1149 |
| 52 | 0.2096 | 0.2588 | 0.1631 | 0.1595 | 0.2324 | 0.2356 | 0.1347 | 0.1315 |
| 53 | 0.2044 | 0.2679 | 0.1644 | 0.1622 | 0.2285 | 0.2340 | 0.1346 | 0.1356 |
| 54 | 0.2008 | 0.2712 | 0.1658 | 0.1586 | 0.2257 | 0.2304 | 0.1340 | 0.1344 |
| 55 | 0.1982 | 0.2703 | 0.1614 | 0.1626 | 0.2233 | 0.2264 | 0.1376 | 0.1284 |
| 56 | 0.1939 | 0.2684 | 0.1548 | 0.1570 | 0.2191 | 0.2203 | 0.1246 | 0.1327 |
| 57 | 0.1881 | 0.2652 | 0.1501 | 0.1442 | 0.2133 | 0.2097 | 0.1233 | 0.1245 |
| 58 | 0.1811 | 0.2542 | 0.1448 | 0.1358 | 0.2054 | 0.2020 | 0.1116 | 0.1237 |
| 59 | 0.1732 | 0.2417 | 0.1320 | 0.1331 | 0.1954 | 0.1858 | 0.1079 | 0.1258 |
| 60 | 0.1640 | 0.2279 | 0.1193 | 0.1268 | 0.1848 | 0.1736 | 0.0988 | 0.1108 |
| 61 | 0.1519 | 0.2183 | 0.1092 | 0.1274 | 0.1710 | 0.1643 | 0.0887 | 0.1030 |
| 62 | 0.1405 | 0.1945 | 0.0994 | 0.1116 | 0.1562 | 0.1499 | 0.0840 | 0.0975 |
| 63 | 0.1259 | 0.1819 | 0.0904 | 0.0989 | 0.1402 | 0.1309 | 0.0707 | 0.0909 |
| 64 | 0.1107 | 0.1585 | 0.0815 | 0.0819 | 0.1213 | 0.1202 | 0.0587 | 0.0828 |
| 65 | 0.0906 | 0.1296 | 0.0675 | 0.0724 | 0.0977 | 0.0964 | 0.0458 | 0.0697 |
| 66 | 0.0746 | 0.1092 | 0.0624 | 0.0581 | 0.0795 | 0.0751 | 0.0410 | 0.0536 |
| 67 | 0.0556 | 0.0811 | 0.0440 | 0.0441 | 0.0562 | 0.0505 | 0.0307 | 0.0363 |
| 68 | 0.0561 | 0.0783 | 0.0445 | 0.0438 | 0.0565 | 0.0496 | 0.0296 | 0.0369 |
| 69 | 0.0563 | 0.0740 | 0.0446 | 0.0480 | 0.0568 | 0.0486 | 0.0295 | 0.0391 |
| 70 | 0.0562 | 0.0738 | 0.0465 | 0.0463 | 0.0567 | 0.0495 | 0.0285 | 0.0406 |
| 71 | 0.0552 | 0.0810 | 0.0514 | 0.0514 | 0.0566 | 0.0501 | 0.0299 | 0.0408 |
| 72 | 0.0548 | 0.0785 | 0.0578 | 0.0533 | 0.0562 | 0.0530 | 0.0298 | 0.0416 |
| 73 | 0.0542 | 0.0802 | 0.0622 | 0.0691 | 0.0556 | 0.0545 | 0.0354 | 0.0442 |
| 74 | 0.0538 | 0.0773 | 0.0622 | 0.0818 | 0.0556 | 0.0562 | 0.0353 | 0.0445 |
| 75 | 0.0535 | 0.0763 | 0.0636 | 0.0866 | 0.0553 | 0.0582 | 0.0365 | 0.0445 |
| 76 | 0.0543 | 0.0733 | 0.0646 | 0.0878 | 0.0549 | 0.0582 | 0.0373 | 0.0546 |
| 77 | 0.0537 | 0.0716 | 0.0696 | 0.0960 | 0.0545 | 0.0614 | 0.0374 | 0.0546 |
| 78 | 0.0544 | 0.0671 | 0.0709 | 0.0960 | 0.0545 | 0.0599 | 0.0379 | 0.0561 |
| 79 | 0.0542 | 0.0673 | 0.0730 | 0.1047 | 0.0544 | 0.0580 | 0.0395 | 0.0561 |
| 80 | 0.0541 | 0.0666 | 0.0761 | 0.1081 | 0.0546 | 0.0566 | 0.0406 | 0.0557 |
| 81 | 0.0546 | 0.0637 | 0.0736 | 0.1241 | 0.0543 | 0.0599 | 0.0390 | 0.0557 |
| 82 | 0.0542 | 0.0614 | 0.0789 | 0.1241 | 0.0543 | 0.0631 | 0.0371 | 0.0542 |
| 83 | 0.0540 | 0.0606 | 0.0804 | 0.1241 | 0.0536 | 0.0660 | 0.0402 | 0.0542 |
| 84 | 0.0537 | 0.0643 | 0.0840 | 0.1298 | 0.0528 | 0.0695 | 0.0417 | 0.0542 |
| 85 | 0.0534 | 0.0636 | 0.0878 | 0.1540 | 0.0530 | 0.0686 | 0.0429 | 0.0581 |
| 86 | 0.0554 | 0.0427 | 0.0826 | 0.1731 | 0.0531 | 0.0673 | 0.0446 | 0.0674 |
| 87 | 0.0564 | 0.0399 | 0.0690 | 0.1731 | 0.0536 | 0.0638 | 0.0455 | 0.0966 |
| 88 | 0.0579 | 0.0399 | 0.0573 | 0.1449 | 0.0547 | 0.0603 | 0.0293 | 0.0966 |
| 89 | 0.0595 | 0.0432 | 0.0748 | 0.0824 | 0.0545 | 0.0600 | 0.0269 | 0.0966 |
| 90+ | 0.0605 | 0.0452 | 0.0876 | 0.0658 | 0.0541 | 0.0576 | 0.0269 | 0.0966 |

| **Initial probabilities of former smoking** | | | | | | | | |
| --- | --- | --- | --- | --- | --- | --- | --- | --- |
|  | **Male** | | | | **Female** | | | |
| **Age** | **White, non-Hispanic** | **Black, non-Hispanic** | **Hispanic** | **Other*** | **White, non-Hispanic** | **Black, non-Hispanic** | **Hispanic** | **Other*** |
| 9 | 0.0000 | 0.0000 | 0.0000 | 0.0000 | 0.0000 | 0.0000 | 0.0000 | 0.0000 |
| 10 | 0.0000 | 0.0000 | 0.0000 | 0.0000 | 0.0000 | 0.0000 | 0.0000 | 0.0000 |
| 11 | 0.0000 | 0.0000 | 0.0000 | 0.0000 | 0.0000 | 0.0000 | 0.0000 | 0.0000 |
| 12 | 0.0000 | 0.0000 | 0.0000 | 0.0000 | 0.0000 | 0.0000 | 0.0000 | 0.0000 |
| 13 | 0.0000 | 0.0000 | 0.0000 | 0.0000 | 0.0000 | 0.0000 | 0.0000 | 0.0000 |
| 14 | 0.0000 | 0.0000 | 0.0000 | 0.0000 | 0.0000 | 0.0000 | 0.0000 | 0.0000 |
| 15 | 0.0000 | 0.0000 | 0.0000 | 0.0000 | 0.0000 | 0.0000 | 0.0000 | 0.0000 |
| 16 | 0.0000 | 0.0000 | 0.0000 | 0.0000 | 0.0000 | 0.0000 | 0.0000 | 0.0000 |
| 17 | 0.0000 | 0.0000 | 0.0000 | 0.0000 | 0.0000 | 0.0000 | 0.0000 | 0.0000 |
| 18 | 0.0265 | 0.0136 | 0.0216 | 0.0225 | 0.0246 | 0.0100 | 0.0176 | 0.0048 |
| 19 | 0.0421 | 0.0237 | 0.0359 | 0.0356 | 0.0385 | 0.0157 | 0.0273 | 0.0103 |
| 20 | 0.0548 | 0.0287 | 0.0453 | 0.0433 | 0.0586 | 0.0232 | 0.0411 | 0.0156 |
| 21 | 0.0709 | 0.0384 | 0.0563 | 0.0514 | 0.0732 | 0.0291 | 0.0496 | 0.0190 |
| 22 | 0.0854 | 0.0459 | 0.0673 | 0.0597 | 0.0901 | 0.0355 | 0.0583 | 0.0223 |
| 23 | 0.0967 | 0.0495 | 0.0757 | 0.0696 | 0.1062 | 0.0404 | 0.0645 | 0.0266 |
| 24 | 0.1115 | 0.0558 | 0.0858 | 0.0773 | 0.1245 | 0.0475 | 0.0723 | 0.0364 |
| 25 | 0.1245 | 0.0617 | 0.0964 | 0.0848 | 0.1271 | 0.0473 | 0.0735 | 0.0363 |
| 26 | 0.1439 | 0.0667 | 0.1129 | 0.0834 | 0.1441 | 0.0428 | 0.0777 | 0.0398 |
| 27 | 0.1591 | 0.0679 | 0.1251 | 0.1000 | 0.1525 | 0.0463 | 0.0787 | 0.0464 |
| 28 | 0.1683 | 0.0733 | 0.1304 | 0.1113 | 0.1579 | 0.0471 | 0.0765 | 0.0508 |
| 29 | 0.1727 | 0.0737 | 0.1373 | 0.1190 | 0.1644 | 0.0433 | 0.0742 | 0.0534 |
| 30 | 0.1881 | 0.0765 | 0.1454 | 0.1185 | 0.1783 | 0.0521 | 0.0796 | 0.0609 |
| 31 | 0.1912 | 0.0871 | 0.1482 | 0.1234 | 0.1790 | 0.0539 | 0.0806 | 0.0610 |
| 32 | 0.1913 | 0.0906 | 0.1480 | 0.1192 | 0.1764 | 0.0535 | 0.0789 | 0.0646 |
| 33 | 0.1976 | 0.0970 | 0.1518 | 0.1236 | 0.1774 | 0.0590 | 0.0802 | 0.0690 |
| 34 | 0.1980 | 0.0911 | 0.1469 | 0.1239 | 0.1712 | 0.0564 | 0.0758 | 0.0663 |
| 35 | 0.1994 | 0.0995 | 0.1493 | 0.1270 | 0.1670 | 0.0566 | 0.0734 | 0.0681 |
| 36 | 0.1970 | 0.0966 | 0.1507 | 0.1351 | 0.1663 | 0.0588 | 0.0751 | 0.0717 |
| 37 | 0.1930 | 0.0921 | 0.1522 | 0.1479 | 0.1666 | 0.0604 | 0.0755 | 0.0740 |
| 38 | 0.1894 | 0.0973 | 0.1518 | 0.1494 | 0.1608 | 0.0609 | 0.0758 | 0.0734 |
| 39 | 0.1907 | 0.1026 | 0.1615 | 0.1565 | 0.1681 | 0.0662 | 0.0814 | 0.0763 |
| 40 | 0.1852 | 0.1026 | 0.1585 | 0.1556 | 0.1732 | 0.0712 | 0.0852 | 0.0819 |
| 41 | 0.1885 | 0.1095 | 0.1712 | 0.1721 | 0.1783 | 0.0778 | 0.0947 | 0.0926 |
| 42 | 0.1887 | 0.1170 | 0.1735 | 0.1708 | 0.1784 | 0.0823 | 0.0942 | 0.0854 |
| 43 | 0.1851 | 0.1213 | 0.1747 | 0.1750 | 0.1920 | 0.0934 | 0.1037 | 0.0922 |
| 44 | 0.1873 | 0.1273 | 0.1738 | 0.1758 | 0.1926 | 0.1011 | 0.1079 | 0.0965 |
| 45 | 0.1952 | 0.1379 | 0.1822 | 0.1876 | 0.1885 | 0.0994 | 0.1067 | 0.0893 |
| 46 | 0.1984 | 0.1371 | 0.1873 | 0.1963 | 0.2001 | 0.1103 | 0.1123 | 0.0975 |
| 47 | 0.2099 | 0.1558 | 0.2011 | 0.1950 | 0.2158 | 0.1250 | 0.1272 | 0.1055 |
| 48 | 0.2256 | 0.1772 | 0.2229 | 0.2166 | 0.2225 | 0.1270 | 0.1322 | 0.1097 |
| 49 | 0.2387 | 0.1903 | 0.2369 | 0.2351 | 0.2318 | 0.1393 | 0.1381 | 0.1111 |
| 50 | 0.2550 | 0.2053 | 0.2526 | 0.2577 | 0.2449 | 0.1545 | 0.1470 | 0.1117 |
| 51 | 0.2657 | 0.2132 | 0.2673 | 0.2745 | 0.2445 | 0.1543 | 0.1464 | 0.1070 |
| 52 | 0.2824 | 0.2319 | 0.2817 | 0.2717 | 0.2406 | 0.1593 | 0.1474 | 0.1077 |
| 53 | 0.2951 | 0.2422 | 0.2944 | 0.2731 | 0.2446 | 0.1670 | 0.1512 | 0.1087 |
| 54 | 0.3129 | 0.2575 | 0.3037 | 0.2933 | 0.2501 | 0.1719 | 0.1594 | 0.1008 |
| 55 | 0.3167 | 0.2523 | 0.3070 | 0.2969 | 0.2566 | 0.1802 | 0.1623 | 0.1025 |
| 56 | 0.3362 | 0.2679 | 0.3116 | 0.2922 | 0.2620 | 0.1899 | 0.1608 | 0.0972 |
| 57 | 0.3565 | 0.2801 | 0.3261 | 0.3122 | 0.2629 | 0.1975 | 0.1643 | 0.1082 |
| 58 | 0.3636 | 0.2896 | 0.3317 | 0.3041 | 0.2664 | 0.2032 | 0.1685 | 0.1073 |
| 59 | 0.3737 | 0.2914 | 0.3478 | 0.3266 | 0.2643 | 0.2056 | 0.1663 | 0.0996 |
| 60 | 0.3952 | 0.3007 | 0.3642 | 0.3329 | 0.2739 | 0.2184 | 0.1653 | 0.0997 |
| 61 | 0.4076 | 0.3196 | 0.3704 | 0.3384 | 0.2771 | 0.2216 | 0.1657 | 0.0972 |
| 62 | 0.4156 | 0.3077 | 0.3724 | 0.3543 | 0.2960 | 0.2411 | 0.1769 | 0.0956 |
| 63 | 0.4417 | 0.3225 | 0.3899 | 0.3738 | 0.3037 | 0.2554 | 0.1740 | 0.0950 |
| 64 | 0.4566 | 0.3387 | 0.4004 | 0.3778 | 0.3246 | 0.2644 | 0.1941 | 0.0984 |
| 65 | 0.4808 | 0.3529 | 0.4265 | 0.3866 | 0.3352 | 0.2740 | 0.2033 | 0.1013 |
| 66 | 0.4823 | 0.3635 | 0.4413 | 0.3974 | 0.3483 | 0.2910 | 0.2088 | 0.1049 |
| 67 | 0.4917 | 0.3939 | 0.4531 | 0.4100 | 0.3431 | 0.2768 | 0.2059 | 0.0968 |
| 68 | 0.5144 | 0.4118 | 0.4725 | 0.4074 | 0.3465 | 0.2738 | 0.2079 | 0.0935 |
| 69 | 0.5373 | 0.4341 | 0.4858 | 0.4179 | 0.3383 | 0.2722 | 0.1997 | 0.0987 |
| 70 | 0.5467 | 0.4637 | 0.4888 | 0.4183 | 0.3340 | 0.2642 | 0.1913 | 0.1090 |
| 71 | 0.5652 | 0.4845 | 0.4874 | 0.4318 | 0.3244 | 0.2657 | 0.1877 | 0.1105 |
| 72 | 0.5656 | 0.4927 | 0.4930 | 0.4171 | 0.3318 | 0.2605 | 0.1828 | 0.1084 |
| 73 | 0.5656 | 0.4893 | 0.5022 | 0.4079 | 0.3307 | 0.2656 | 0.1757 | 0.1065 |
| 74 | 0.5585 | 0.4895 | 0.4864 | 0.3704 | 0.3372 | 0.2668 | 0.1731 | 0.1227 |
| 75 | 0.5579 | 0.4915 | 0.4793 | 0.3664 | 0.3350 | 0.2601 | 0.1735 | 0.1439 |
| 76 | 0.5673 | 0.4993 | 0.4941 | 0.3645 | 0.3361 | 0.2587 | 0.1820 | 0.1456 |
| 77 | 0.5995 | 0.5405 | 0.5138 | 0.3744 | 0.3321 | 0.2560 | 0.1840 | 0.1652 |
| 78 | 0.6029 | 0.5346 | 0.5291 | 0.3740 | 0.3252 | 0.2483 | 0.1856 | 0.1576 |
| 79 | 0.6049 | 0.5406 | 0.5390 | 0.3643 | 0.3127 | 0.2465 | 0.1713 | 0.1583 |
| 80 | 0.6112 | 0.5505 | 0.5483 | 0.3735 | 0.3039 | 0.2362 | 0.1606 | 0.1601 |
| 81 | 0.6271 | 0.5680 | 0.5382 | 0.3401 | 0.2979 | 0.2272 | 0.1575 | 0.1619 |
| 82 | 0.6120 | 0.5341 | 0.5138 | 0.3178 | 0.2873 | 0.2250 | 0.1511 | 0.1659 |
| 83 | 0.5968 | 0.5263 | 0.4975 | 0.3281 | 0.2724 | 0.2207 | 0.1442 | 0.1685 |
| 84 | 0.5919 | 0.5234 | 0.5024 | 0.3113 | 0.2615 | 0.2083 | 0.1375 | 0.1693 |
| 85 | 0.5748 | 0.5080 | 0.4903 | 0.2960 | 0.2480 | 0.2074 | 0.1363 | 0.1543 |
| 86 | 0.5461 | 0.4847 | 0.4949 | 0.2816 | 0.2397 | 0.1856 | 0.1273 | 0.1473 |
| 87 | 0.5365 | 0.4685 | 0.4804 | 0.2876 | 0.2288 | 0.1896 | 0.1326 | 0.1461 |
| 88 | 0.5238 | 0.4511 | 0.4468 | 0.2930 | 0.2191 | 0.1682 | 0.1428 | 0.1502 |
| 89 | 0.5097 | 0.4261 | 0.4413 | 0.2909 | 0.2084 | 0.1585 | 0.1437 | 0.1327 |
| 90+ | 0.4911 | 0.4047 | 0.4477 | 0.3117 | 0.1985 | 0.1591 | 0.1602 | 0.1003 |

## Incident smoking-attributable disease cases and mortality rates

| **Incident cases per 100,000 by disease, age, sex and smoking status** | | | | | | |
| --- | --- | --- | --- | --- | --- | --- |
| **Cancer of the Oral Cavity and Pharynx*** | | | | | | |
|  | Males | | | Females | | |
| Age* | Never | Current | Former | Never | Current | Former |
| 35-39 | 3.0 | 5.2 | 4.1 | 2.4 | 3.0 | 2.9 |
| 40-44 | 6.7 | 11.6 | 9.1 | 3.1 | 3.9 | 3.8 |
| 45-49 | 13.5 | 23.4 | 18.3 | 5.6 | 7.2 | 6.9 |
| 50-54 | 23.6 | 41.0 | 32.1 | 8.7 | 11.1 | 10.8 |
| 55-59 | 35.0 | 65.2 | 45.9 | 9.9 | 20.6 | 12.7 |
| 60-64 | 42.0 | 78.1 | 55.0 | 12.8 | 26.6 | 16.4 |
| 65-69 | 42.4 | 99.6 | 63.2 | 16.1 | 33.1 | 20.2 |
| 70-74 | 44.1 | 103.6 | 65.7 | 20.5 | 42.2 | 25.8 |
| 75-79 | 47.4 | 103.4 | 69.2 | 23.9 | 46.1 | 30.3 |
| 80-84 | 49.9 | 108.8 | 72.8 | 25.5 | 49.2 | 32.4 |
| 85+ | 48.6 | 105.9 | 70.9 | 29.2 | 56.4 | 37.1 |
| **Cancer of the Esophagus*** | | | | | | |
|  | Males | | | Females | | |
| Age* | Never | Current | Former | Never | Current | Former |
| 35-39 | 0.4 | 0.7 | 0.6 | 0.2 | 0.2 | 0.2 |
| 40-44 | 1.1 | 1.9 | 1.5 | 0.4 | 0.5 | 0.4 |
| 45-49 | 3.1 | 5.5 | 4.3 | 0.6 | 0.8 | 0.8 |
| 50-54 | 6.0 | 10.5 | 8.2 | 1.6 | 2.1 | 2.0 |
| 55-59 | 10.8 | 20.1 | 14.2 | 2.4 | 5.0 | 3.1 |
| 60-64 | 18.4 | 34.2 | 24.1 | 3.3 | 6.8 | 4.2 |
| 65-69 | 21.7 | 51.0 | 32.4 | 5.0 | 10.3 | 6.3 |
| 70-74 | 25.3 | 59.5 | 37.7 | 7.3 | 15.0 | 9.2 |
| 75-79 | 32.5 | 70.8 | 47.4 | 9.0 | 17.4 | 11.4 |
| 80-84 | 33.3 | 72.6 | 48.6 | 11.6 | 22.3 | 14.7 |
| 85+ | 32.6 | 71.1 | 47.6 | 12.0 | 23.2 | 15.3 |
| **Cancer of the Stomach*** | | | | | | |
|  | Males | | | Females | | |
| Age* | Never | Current | Former | Never | Current | Former |
| 35-39 | 1.5 | 2.6 | 2.0 | 1.6 | 2.1 | 2.0 |
| 40-44 | 2.7 | 4.7 | 3.7 | 2.6 | 3.4 | 3.2 |
| 45-49 | 4.8 | 8.4 | 6.5 | 3.6 | 4.6 | 4.5 |
| 50-54 | 7.9 | 13.7 | 10.7 | 5.3 | 6.8 | 6.5 |
| 55-59 | 11.7 | 21.8 | 15.4 | 5.9 | 12.3 | 7.6 |
| 60-64 | 18.0 | 33.5 | 23.6 | 8.5 | 17.6 | 10.8 |
| 65-69 | 24.2 | 56.9 | 36.1 | 12.9 | 26.7 | 16.3 |
| 70-74 | 33.4 | 78.5 | 49.8 | 20.0 | 41.2 | 25.2 |
| 75-79 | 44.7 | 97.5 | 65.3 | 25.5 | 49.3 | 32.4 |
| 80-84 | 53.4 | 116.5 | 78.0 | 33.1 | 63.8 | 42.0 |
| 85+ | 59.6 | 130.0 | 87.1 | 36.1 | 69.6 | 45.8 |
|  |  |  |  |  |  |  |
|  |  |  |  |  |  |  |
| **Incident cases per 100,000 by disease, age, sex and smoking status (Continued)** | | | | | | |
| **Cancer of the Colon and Rectum*** | | | | | | |
|  | Males | | | Females | | |
| Age* | Never | Current | Former | Never | Current | Former |
| 35-39 | 7.6 | 13.1 | 10.3 | 8.1 | 10.3 | 10.0 |
| 40-44 | 14.2 | 24.7 | 19.3 | 15.1 | 19.3 | 18.7 |
| 45-49 | 26.4 | 45.9 | 35.9 | 26.2 | 33.5 | 32.5 |
| 50-54 | 50.1 | 87.2 | 68.1 | 45.7 | 58.5 | 56.7 |
| 55-59 | 62.3 | 116.0 | 81.7 | 44.7 | 92.9 | 57.2 |
| 60-64 | 87.3 | 162.3 | 114.3 | 60.8 | 126.6 | 77.9 |
| 65-69 | 111.3 | 261.5 | 165.8 | 88.7 | 182.7 | 111.8 |
| 70-74 | 145.9 | 342.8 | 217.3 | 128.5 | 264.7 | 161.9 |
| 75-79 | 186.3 | 406.2 | 272.0 | 168.3 | 324.8 | 213.7 |
| 80-84 | 232.3 | 506.4 | 339.1 | 222.6 | 429.5 | 282.6 |
| 85+ | 258.8 | 564.3 | 377.9 | 250.2 | 483.0 | 317.8 |
| **Cancer of the Liver and Intrahepatic Bile Duct*** | | | | | | |
|  | Males | | | Females | | |
| Age* | Never | Current | Former | Never | Current | Former |
| 35-39 | 1.0 | 1.7 | 1.4 | 0.5 | 0.7 | 0.7 |
| 40-44 | 2.1 | 3.6 | 2.8 | 1.0 | 1.3 | 1.2 |
| 45-49 | 6.5 | 11.2 | 8.8 | 2.1 | 2.7 | 2.6 |
| 50-54 | 17.4 | 30.3 | 23.7 | 4.6 | 6.0 | 5.8 |
| 55-59 | 34.3 | 63.7 | 44.9 | 7.7 | 16.1 | 9.9 |
| 60-64 | 39.3 | 73.1 | 51.5 | 9.9 | 20.6 | 12.7 |
| 65-69 | 32.2 | 75.6 | 47.9 | 12.9 | 26.5 | 16.2 |
| 70-74 | 34.6 | 81.3 | 51.5 | 17.8 | 36.7 | 22.4 |
| 75-79 | 40.3 | 87.8 | 58.8 | 21.5 | 41.4 | 27.3 |
| 80-84 | 43.8 | 95.5 | 64.0 | 24.8 | 47.8 | 31.5 |
| 85+ | 36.9 | 80.5 | 53.9 | 21.7 | 41.8 | 27.5 |
| **Cancer of the Pancreas*** | | | | | | |
|  | Males | | | Females | | |
| Age* | Never | Current | Former | Never | Current | Former |
| 35-39 | 1.1 | 1.9 | 1.5 | 0.9 | 1.2 | 1.1 |
| 40-44 | 2.2 | 3.9 | 3.0 | 2.2 | 2.8 | 2.7 |
| 45-49 | 5.0 | 8.8 | 6.8 | 4.3 | 5.5 | 5.4 |
| 50-54 | 10.2 | 17.7 | 13.8 | 7.9 | 10.1 | 9.8 |
| 55-59 | 17.6 | 32.7 | 23.0 | 12.2 | 25.3 | 15.6 |
| 60-64 | 28.5 | 52.9 | 37.3 | 20.0 | 41.7 | 25.7 |
| 65-69 | 36.8 | 86.5 | 54.9 | 31.3 | 64.5 | 39.4 |
| 70-74 | 48.5 | 114.0 | 72.3 | 46.7 | 96.2 | 58.8 |
| 75-79 | 64.1 | 139.8 | 93.6 | 62.7 | 121.1 | 79.7 |
| 80-84 | 79.7 | 173.7 | 116.3 | 79.8 | 154.0 | 101.3 |
| 85+ | 84.1 | 183.2 | 122.7 | 89.2 | 172.2 | 113.3 |
|  |  |  |  |  |  |  |
|  |  |  |  |  |  |  |
|  |  |  |  |  |  |  |
| **Incident cases per 100,000 by disease, age, sex and smoking status (Continued)** | | | | | | |
| **Cancer of the Larynx*** | | | | | | |
|  | Males | | | Females | | |
| Age* | Never | Current | Former | Never | Current | Former |
| 35-39 | 0.4 | 0.7 | 0.6 | 0.2 | 0.2 | 0.2 |
| 40-44 | 1.0 | 1.8 | 1.4 | 0.4 | 0.5 | 0.4 |
| 45-49 | 2.6 | 4.5 | 3.5 | 1.0 | 1.3 | 1.2 |
| 50-54 | 5.7 | 9.9 | 7.8 | 2.1 | 2.6 | 2.5 |
| 55-59 | 9.3 | 17.4 | 12.2 | 2.3 | 4.9 | 3.0 |
| 60-64 | 14.5 | 27.0 | 19.0 | 2.8 | 5.8 | 3.6 |
| 65-69 | 16.7 | 39.3 | 24.9 | 3.8 | 7.8 | 4.7 |
| 70-74 | 20.1 | 47.1 | 29.9 | 4.5 | 9.3 | 5.7 |
| 75-79 | 22.0 | 48.0 | 32.1 | 4.6 | 8.9 | 5.8 |
| 80-84 | 22.0 | 48.0 | 32.2 | 3.4 | 6.5 | 4.3 |
| 85+ | 18.7 | 40.7 | 27.2 | 2.8 | 5.4 | 3.6 |
| **Cancer of the Lung and Bronchus*** | | | | | | |
|  | Males | | | Females | | |
| Age* | Never | Current | Former | Never | Current | Former |
| 35-39 | 0.7 | 9.5 | 2.9 | 0.7 | 9.5 | 1.9 |
| 40-44 | 1.3 | 18.9 | 5.8 | 2.1 | 28.0 | 5.6 |
| 45-49 | 3.9 | 56.5 | 17.3 | 5.6 | 75.0 | 14.9 |
| 50-54 | 9.3 | 133.2 | 40.9 | 11.6 | 154.6 | 30.7 |
| 55-59 | 14.9 | 284.3 | 68.3 | 13.4 | 253.1 | 66.8 |
| 60-64 | 26.4 | 502.6 | 120.7 | 23.7 | 449.3 | 118.6 |
| 65-69 | 32.9 | 932.0 | 256.6 | 36.0 | 852.1 | 245.0 |
| 70-74 | 48.2 | 1,364.3 | 375.7 | 66.0 | 1,561.6 | 449.0 |
| 75-79 | 87.1 | 1,959.9 | 562.5 | 77.9 | 1,797.1 | 496.8 |
| 80-84 | 107.3 | 2,415.7 | 693.3 | 110.6 | 2,553.6 | 705.9 |
| 85+ | 90.5 | 2,037.1 | 584.6 | 80.3 | 1,852.2 | 512.0 |
| **Cancer of the Cervix Uteri*** | | | | | | |
|  | Males | | | Females | | |
| Age* | Never | Current | Former | Never | Current | Former |
| 35-39 | - | - | - | 12.1 | 15.4 | 14.9 |
| 40-44 | - | - | - | 13.2 | 16.9 | 16.4 |
| 45-49 | - | - | - | 11.9 | 15.3 | 14.8 |
| 50-54 | - | - | - | 11.0 | 14.1 | 13.6 |
| 55-59 | - | - | - | 9.1 | 18.8 | 11.6 |
| 60-64 | - | - | - | 9.7 | 20.1 | 12.4 |
| 65-69 | - | - | - | 9.7 | 20.1 | 12.3 |
| 70-74 | - | - | - | 10.2 | 21.0 | 12.9 |
| 75-79 | - | - | - | 8.7 | 16.7 | 11.0 |
| 80-84 | - | - | - | 8.7 | 16.9 | 11.1 |
| 85+ | - | - | - | 8.5 | 16.3 | 10.8 |
|  |  |  |  |  |  |  |
|  |  |  |  |  |  |  |
|  |  |  |  |  |  |  |
| **Incident cases per 100,000 by disease, age, sex and smoking status (Continued)** | | | | | | |
| **CanceroftheUrinaryBladder (InvasiveandInSitu)*** | | | | | | |
|  | Males | | | Females | | |
| Age* | Never | Current | Former | Never | Current | Former |
| 35-39 | 1.6 | 2.7 | 2.1 | 0.7 | 0.9 | 0.9 |
| 40-44 | 3.3 | 5.8 | 4.5 | 1.5 | 2.0 | 1.9 |
| 45-49 | 7.3 | 12.7 | 10.0 | 2.8 | 3.6 | 3.5 |
| 50-54 | 14.8 | 25.7 | 20.1 | 5.7 | 7.3 | 7.1 |
| 55-59 | 29.2 | 54.4 | 38.3 | 8.4 | 17.5 | 10.8 |
| 60-64 | 52.6 | 97.8 | 68.9 | 15.2 | 31.6 | 19.4 |
| 65-69 | 85.7 | 201.5 | 127.8 | 25.0 | 51.5 | 31.5 |
| 70-74 | 124.3 | 292.2 | 185.3 | 36.4 | 75.0 | 45.9 |
| 75-79 | 189.2 | 412.5 | 276.3 | 50.8 | 98.0 | 64.5 |
| 80-84 | 250.6 | 546.3 | 365.9 | 64.2 | 123.9 | 81.5 |
| 85+ | 284.3 | 619.8 | 415.1 | 69.8 | 134.8 | 88.7 |
| **CanceroftheKidneyandRenalPelvis*** | | | | | | |
|  | Males | | | Females | | |
| Age* | Never | Current | Former | Never | Current | Former |
| 35-39 | 5.2 | 9.1 | 7.1 | 3.7 | 4.8 | 4.6 |
| 40-44 | 9.7 | 16.9 | 13.2 | 6.1 | 7.7 | 7.5 |
| 45-49 | 15.7 | 27.3 | 21.3 | 9.1 | 11.7 | 11.3 |
| 50-54 | 23.0 | 40.1 | 31.3 | 13.3 | 17.1 | 16.5 |
| 55-59 | 33.9 | 63.0 | 44.4 | 16.2 | 33.6 | 20.7 |
| 60-64 | 46.4 | 86.4 | 60.8 | 22.4 | 46.5 | 28.6 |
| 65-69 | 58.1 | 136.6 | 86.6 | 32.5 | 67.0 | 41.0 |
| 70-74 | 66.3 | 155.9 | 98.8 | 39.4 | 81.2 | 49.7 |
| 75-79 | 74.2 | 161.7 | 108.3 | 43.1 | 83.2 | 54.7 |
| 80-84 | 75.7 | 165.0 | 110.5 | 43.1 | 83.1 | 54.7 |
| 85+ | 62.9 | 137.0 | 91.8 | 36.3 | 70.1 | 46.1 |
| **Acute Myeloid Leukemia*** | | | | | | |
|  | Males | | | Females | | |
| Age* | Never | Current | Former | Never | Current | Former |
| 35-39 | 1.4 | 2.5 | 1.9 | 1.4 | 1.7 | 1.7 |
| 40-44 | 1.6 | 2.8 | 2.2 | 1.7 | 2.2 | 2.1 |
| 45-49 | 1.9 | 3.3 | 2.6 | 2.3 | 2.9 | 2.8 |
| 50-54 | 2.9 | 5.0 | 3.9 | 2.9 | 3.7 | 3.5 |
| 55-59 | 4.1 | 7.6 | 5.4 | 3.1 | 6.5 | 4.0 |
| 60-64 | 7.1 | 13.2 | 9.3 | 5.1 | 10.6 | 6.5 |
| 65-69 | 10.1 | 23.7 | 15.0 | 7.9 | 16.2 | 9.9 |
| 70-74 | 14.8 | 34.8 | 22.1 | 11.8 | 24.2 | 14.8 |
| 75-79 | 21.9 | 47.8 | 32.0 | 14.2 | 27.4 | 18.0 |
| 80-84 | 28.6 | 62.2 | 41.7 | 18.4 | 35.5 | 23.4 |
| 85+ | 28.5 | 62.1 | 41.6 | 17.6 | 33.9 | 22.3 |
|  |  |  |  |  |  |  |
|  |  |  |  |  |  |  |
|  |  |  |  |  |  |  |
| **Incident cases per 100,000 by disease, age, sex and smoking status (Continued)** | | | | | | |
| **Ischemic Heart Disease*** | | | | | | |
|  | Males | | | Females | | |
| Age* | Never | Current | Former | Never | Current | Former |
| 35-39 | 87.2 | 338.3 | 159.6 | 39.4 | 196.1 | 87.8 |
| 40-44 | 79.3 | 307.7 | 145.1 | 40.3 | 200.8 | 89.9 |
| 45-49 | 358.7 | 1,391.7 | 656.4 | 109.8 | 546.7 | 244.8 |
| 50-54 | 350.7 | 1,360.9 | 641.9 | 107.0 | 533.0 | 238.7 |
| 55-59 | 724.8 | 2,167.1 | 1,101.7 | 379.5 | 1,233.4 | 459.2 |
| 60-64 | 728.2 | 2,177.2 | 1,106.8 | 392.4 | 1,275.3 | 474.8 |
| 65-69 | 1,388.5 | 3,832.3 | 2,193.8 | 662.8 | 2,180.7 | 1,034.0 |
| 70-74 | 1,398.7 | 3,860.3 | 2,209.9 | 731.8 | 2,407.6 | 1,141.6 |
| 75-79 | 2,281.8 | 4,518.0 | 3,012.0 | 1,458.6 | 3,281.9 | 2,071.2 |
| 80-84 | 2,343.2 | 4,639.5 | 3,093.0 | 1,559.4 | 3,508.6 | 2,214.3 |
| 85+ | 2,668.6 | 5,283.8 | 3,522.5 | 2,222.8 | 5,001.2 | 3,156.3 |
| **Other Heart Disease*** | | | | | | |
|  | Males | | | Females | | |
| Age* | Never | Current | Former | Never | Current | Former |
| 35-39 | 164.7 | 395.3 | 176.2 | 129.4 | 315.6 | 129.4 |
| 40-44 | 156.8 | 376.3 | 167.8 | 133.2 | 325.1 | 133.2 |
| 45-49 | 467.0 | 1,120.9 | 499.7 | 294.4 | 718.3 | 294.4 |
| 50-54 | 472.4 | 1,133.8 | 505.5 | 294.9 | 719.5 | 294.9 |
| 55-59 | 781.9 | 1,962.6 | 1,180.7 | 689.2 | 1,364.7 | 758.1 |
| 60-64 | 779.5 | 1,956.6 | 1,177.1 | 701.6 | 1,389.2 | 771.8 |
| 65-69 | 1,876.5 | 4,165.7 | 2,476.9 | 1,643.2 | 3,039.9 | 2,119.7 |
| 70-74 | 1,894.1 | 4,205.0 | 2,500.3 | 1,727.5 | 3,196.0 | 2,228.5 |
| 75-79 | 4,524.5 | 7,510.7 | 5,203.2 | 3,788.2 | 6,629.3 | 5,000.4 |
| 80-84 | 4,602.7 | 7,640.4 | 5,293.1 | 3,957.7 | 6,926.0 | 5,224.2 |
| 85+ | 8,192.8 | 13,600.0 | 9,421.7 | 6,563.0 | 11,485.2 | 8,663.1 |
| **Cerobrovascular Disease*** | | | | | | |
|  | Males | | | Females | | |
| Age* | Never | Current | Former | Never | Current | Former |
| 35-39 | 88.5 | 212.3 | 94.7 | 45.6 | 111.3 | 45.6 |
| 40-44 | 84.2 | 202.1 | 90.1 | 47.0 | 114.7 | 47.0 |
| 45-49 | 198.2 | 475.6 | 212.0 | 179.4 | 437.7 | 179.4 |
| 50-54 | 200.4 | 481.0 | 214.5 | 179.7 | 438.4 | 179.7 |
| 55-59 | 410.2 | 1,029.7 | 619.5 | 334.2 | 661.6 | 367.6 |
| 60-64 | 409.0 | 1,026.6 | 617.6 | 340.2 | 673.5 | 374.2 |
| 65-69 | 945.7 | 2,052.2 | 1,163.3 | 794.0 | 1,802.4 | 984.6 |
| 70-74 | 957.9 | 2,078.7 | 1,178.3 | 842.2 | 1,911.8 | 1,044.4 |
| 75-79 | 1,980.2 | 2,930.7 | 2,217.8 | 1,624.0 | 2,760.7 | 1,786.4 |
| 80-84 | 2,006.5 | 2,969.6 | 2,247.3 | 1,686.5 | 2,867.1 | 1,855.2 |
| 85+ | 2,327.3 | 3,444.4 | 2,606.5 | 3,223.6 | 5,480.1 | 3,545.9 |
|  |  |  |  |  |  |  |
|  |  |  |  |  |  |  |
|  |  |  |  |  |  |  |
| **Incident cases per 100,000 by disease, age, sex and smoking status (Continued)** | | | | | | |
| **Other Vascular Diseases*** | | | | | | |
|  | Males | | | Females | | |
| Age* | Never | Current | Former | Never | Current | Former |
| 35-39 | 21.5 | 51.6 | 23.0 | 24.6 | 60.1 | 24.6 |
| 40-44 | 20.5 | 49.1 | 21.9 | 25.4 | 61.9 | 25.4 |
| 45-49 | 40.4 | 97.0 | 43.2 | 30.4 | 74.2 | 30.4 |
| 50-54 | 40.9 | 98.1 | 43.7 | 30.4 | 74.3 | 30.4 |
| 55-59 | 125.0 | 313.7 | 188.7 | 107.0 | 211.8 | 117.7 |
| 60-64 | 124.6 | 312.7 | 188.1 | 108.9 | 215.7 | 119.8 |
| 65-69 | 238.5 | 1,729.0 | 524.7 | 93.5 | 636.6 | 211.3 |
| 70-74 | 247.2 | 1,792.4 | 543.9 | 110.9 | 755.2 | 250.6 |
| 75-79 | 434.7 | 2,143.3 | 747.8 | 244.6 | 1,411.4 | 494.1 |
| 80-84 | 464.0 | 2,287.8 | 798.2 | 293.4 | 1,692.8 | 592.6 |
| 85+ | 604.8 | 2,981.5 | 1,040.2 | 407.7 | 2,352.4 | 823.5 |
| **Diabetes*** | | | | | | |
|  | Males | | | Females | | |
| Age* | Never | Current | Former | Never | Current | Former |
| 35-39 | 96.5 | 231.7 | 103.3 | 101.8 | 248.3 | 101.8 |
| 40-44 | 91.9 | 220.6 | 98.3 | 104.8 | 255.8 | 104.8 |
| 45-49 | 238.1 | 571.5 | 254.8 | 220.1 | 537.0 | 220.1 |
| 50-54 | 240.8 | 578.0 | 257.7 | 220.4 | 537.9 | 220.4 |
| 55-59 | 270.8 | 679.7 | 408.9 | 282.7 | 559.8 | 311.0 |
| 60-64 | 270.0 | 677.6 | 407.7 | 287.8 | 569.9 | 316.6 |
| 65-69 | 330.5 | 495.7 | 505.6 | 304.5 | 468.9 | 392.8 |
| 70-74 | 326.1 | 489.1 | 498.9 | 317.2 | 488.4 | 409.1 |
| 75-79 | 674.9 | 674.9 | 715.4 | 479.2 | 527.1 | 508.0 |
| 80-84 | 676.6 | 676.6 | 717.2 | 482.5 | 530.8 | 511.5 |
| 85+ | 561.1 | 561.1 | 594.7 | 664.1 | 730.6 | 704.0 |
| **Influenza, Pneumonia*** | | | | | | |
|  | Males | | | Females | | |
| Age* | Never | Current | Former | Never | Current | Former |
| 35-39 | 41.8 | 187.1 | 92.9 | 45.9 | 295.0 | 84.9 |
| 40-44 | 37.5 | 167.8 | 83.4 | 48.0 | 308.9 | 88.9 |
| 45-49 | 127.6 | 570.3 | 283.2 | 107.8 | 693.4 | 199.5 |
| 50-54 | 123.2 | 550.8 | 273.6 | 106.3 | 683.6 | 196.7 |
| 55-59 | 80.8 | 1,225.8 | 321.6 | 114.6 | 1,031.7 | 554.8 |
| 60-64 | 82.7 | 1,254.4 | 329.1 | 123.8 | 1,114.4 | 599.3 |
| 65-69 | 589.3 | 1,520.5 | 954.7 | 647.4 | 1,132.9 | 828.6 |
| 70-74 | 591.0 | 1,524.8 | 957.4 | 678.2 | 1,186.8 | 868.0 |
| 75-79 | 1,460.3 | 2,365.6 | 2,073.6 | 1,501.6 | 3,093.4 | 1,817.0 |
| 80-84 | 1,495.5 | 2,422.7 | 2,123.6 | 1,588.3 | 3,271.8 | 1,921.8 |
| 85+ | 3,908.0 | 6,330.9 | 5,549.3 | 2,946.5 | 6,069.8 | 3,565.3 |
|  |  |  |  |  |  |  |
|  |  |  |  |  |  |  |
|  |  |  |  |  |  |  |
|  |  |  |  |  |  |  |
|  |  |  |  |  |  |  |
| **Incident cases per 100,000 by disease, age, sex and smoking status (Continued)** | | | | | | |
| **Chronic Obstructive Pulmonary Disease*** | | | | | | |
|  | Males | | | Females | | |
| Age* | Never | Current | Former | Never | Current | Former |
| 35-39 | 18.1 | 80.9 | 40.2 | 21.9 | 141.0 | 40.6 |
| 40-44 | 16.2 | 72.6 | 36.1 | 23.0 | 147.7 | 42.5 |
| 45-49 | 59.9 | 267.8 | 133.0 | 99.7 | 641.3 | 184.5 |
| 50-54 | 57.9 | 258.7 | 128.5 | 98.3 | 632.2 | 181.9 |
| 55-59 | 70.2 | 1,064.6 | 279.3 | 118.2 | 1,063.7 | 572.1 |
| 60-64 | 71.8 | 1,089.4 | 285.8 | 127.7 | 1,149.0 | 617.9 |
| 65-69 | 550.9 | 1,421.3 | 892.4 | 867.6 | 1,518.2 | 1,110.5 |
| 70-74 | 552.4 | 1,425.3 | 895.0 | 908.8 | 1,590.5 | 1,163.3 |
| 75-79 | 1,191.5 | 1,930.2 | 1,691.9 | 979.1 | 2,016.9 | 1,184.7 |
| 80-84 | 1,220.2 | 1,976.8 | 1,732.7 | 1,035.5 | 2,133.2 | 1,253.0 |
| 85+ | 1,425.9 | 2,309.9 | 2,024.8 | 1,201.0 | 2,474.1 | 1,453.2 |

*IMPORTANT: The granular age strata and disease categories shown in this table are used in the model to obtain more precise estimates of life years and quality adjusted life years. However, the rates for an individual age group or disease category may be inaccurate by themselves because the underlying relative risks by smoking status are based on broader age groups and combined disease categories. Some of the estimates shown in this table are obtained by applying a relative risk for a broader age group and combined disease categories to more granular age groups and disease categories encompassed by the relevant relative risk estimate. For this reason, model results are reported only for broader categories and the estimates shown in this table should not be used or reported by themselves. For the age strata and disease groups on which the relative risks are estimated, please see U.S. Department of Health and Human Services. The Health Consequences of Smoking—50 Years of Progress: A Report of the Surgeon General, 2014. Atlanta, GA: U.S. Department of Health and Human Services, Centers for Disease Control and Prevention, National Center for Chronic Disease Prevention and Health Promotion, Office on Smoking and Health; 2014.

| **Probability of disease-specific mortality by age, sex, and smoking status, conditional on having a disease event** | | | | | | |
| --- | --- | --- | --- | --- | --- | --- |
| **Cancer of the Oral Cavity and Pharynx*** | | | | | | |
|  | Males | | | Females | | |
| Age* | Never | Current | Former | Never | Current | Former |
| 35-39 | 0.31 | 0.31 | 0.31 | - | - | - |
| 40-44 | 0.13 | 0.13 | 0.13 | - | - | - |
| 45-49 | 0.20 | 0.20 | 0.20 | 0.27 | 0.27 | 0.27 |
| 50-54 | 0.11 | 0.11 | 0.11 | 0.18 | 0.18 | 0.18 |
| 55-59 | 0.19 | 0.19 | 0.19 | 0.29 | 0.29 | 0.29 |
| 60-64 | 0.16 | 0.16 | 0.16 | 0.23 | 0.23 | 0.23 |
| 65-69 | 0.30 | 0.30 | 0.30 | 0.36 | 0.36 | 0.36 |
| 70-74 | 0.29 | 0.29 | 0.29 | 0.30 | 0.30 | 0.30 |
| 75-79 | 0.23 | 0.23 | 0.23 | 0.32 | 0.32 | 0.32 |
| 80-84 | 0.22 | 0.22 | 0.22 | 0.31 | 0.31 | 0.31 |
| 85+ | 0.42 | 0.42 | 0.42 | 0.39 | 0.39 | 0.39 |
| **Cancer of the Esophagus*** | | | | | | |
|  | Males | | | Females | | |
| Age* | Never | Current | Former | Never | Current | Former |
| 35-39 | 1.00 | 1.00 | 1.00 | - | - | - |
| 40-44 | 0.86 | 0.86 | 0.86 | - | - | - |
| 45-49 | 1.00 | 1.00 | 1.00 | - | - | - |
| 50-54 | 0.65 | 0.65 | 0.65 | - | - | - |
| 55-59 | 1.00 | 1.00 | 1.00 | 1.00 | 1.00 | 1.00 |
| 60-64 | 0.66 | 0.66 | 0.66 | 0.78 | 0.78 | 0.78 |
| 65-69 | 1.00 | 1.00 | 1.00 | 1.00 | 1.00 | 1.00 |
| 70-74 | 0.98 | 0.98 | 0.98 | 0.86 | 0.86 | 0.86 |
| 75-79 | 1.00 | 1.00 | 1.00 | 1.00 | 1.00 | 1.00 |
| 80-84 | 1.00 | 1.00 | 1.00 | 1.00 | 1.00 | 1.00 |
| 85+ | 1.00 | 1.00 | 1.00 | 1.00 | 1.00 | 1.00 |
| **Cancer of the Stomach*** | | | | | | |
|  | Males | | | Females | | |
| Age* | Never | Current | Former | Never | Current | Former |
| 35-39 | 0.56 | 0.56 | 0.56 | - | - | - |
| 40-44 | 0.29 | 0.29 | 0.29 | - | - | - |
| 45-49 | 0.51 | 0.51 | 0.51 | - | - | - |
| 50-54 | 0.30 | 0.30 | 0.30 | - | - | - |
| 55-59 | 0.55 | 0.55 | 0.55 | 0.58 | 0.58 | 0.58 |
| 60-64 | 0.35 | 0.35 | 0.35 | 0.42 | 0.42 | 0.42 |
| 65-69 | 0.50 | 0.50 | 0.50 | 0.63 | 0.63 | 0.63 |
| 70-74 | 0.36 | 0.36 | 0.36 | 0.43 | 0.43 | 0.43 |
| 75-79 | 0.72 | 0.72 | 0.72 | 0.57 | 0.57 | 0.57 |
| 80-84 | 0.62 | 0.62 | 0.62 | 0.47 | 0.47 | 0.47 |
| 85+ | 1.00 | 1.00 | 1.00 | 0.82 | 0.82 | 0.82 |
| **Probability of disease-specific mortality by age, sex, and smoking status, conditional on having a disease event (Continued)** | | | | | | |
| **Cancer of the Colon and Rectum*** | | | | | | |
|  | Males | | | Females | | |
| Age* | Never | Current | Former | Never | Current | Former |
| 35-39 | 0.25 | 0.25 | 0.25 | 0.24 | 0.24 | 0.24 |
| 40-44 | 0.13 | 0.13 | 0.13 | 0.13 | 0.13 | 0.13 |
| 45-49 | 0.32 | 0.32 | 0.32 | 0.28 | 0.28 | 0.28 |
| 50-54 | 0.16 | 0.16 | 0.16 | 0.16 | 0.16 | 0.16 |
| 55-59 | 0.50 | 0.50 | 0.50 | 0.47 | 0.47 | 0.47 |
| 60-64 | 0.36 | 0.36 | 0.36 | 0.35 | 0.35 | 0.35 |
| 65-69 | 0.56 | 0.56 | 0.56 | 0.56 | 0.56 | 0.56 |
| 70-74 | 0.43 | 0.43 | 0.43 | 0.41 | 0.41 | 0.41 |
| 75-79 | 0.74 | 0.74 | 0.74 | 0.59 | 0.59 | 0.59 |
| 80-84 | 0.61 | 0.61 | 0.61 | 0.47 | 0.47 | 0.47 |
| 85+ | 0.95 | 0.95 | 0.95 | 0.90 | 0.90 | 0.90 |
| **Cancer of the Liver and Intrahepatic Bile Duct*** | | | | | | |
|  | Males | | | Females | | |
| Age* | Never | Current | Former | Never | Current | Former |
| 35-39 | 0.92 | 0.92 | 0.92 | - | - | - |
| 40-44 | 0.42 | 0.42 | 0.42 | - | - | - |
| 45-49 | 0.45 | 0.45 | 0.45 | 0.57 | 0.57 | 0.57 |
| 50-54 | 0.16 | 0.16 | 0.16 | 0.25 | 0.25 | 0.25 |
| 55-59 | 0.15 | 0.15 | 0.15 | 0.46 | 0.46 | 0.46 |
| 60-64 | 0.13 | 0.13 | 0.13 | 0.37 | 0.37 | 0.37 |
| 65-69 | 0.51 | 0.51 | 0.51 | 0.50 | 0.50 | 0.50 |
| 70-74 | 0.48 | 0.48 | 0.48 | 0.38 | 0.38 | 0.38 |
| 75-79 | 0.58 | 0.58 | 0.58 | 0.69 | 0.69 | 0.69 |
| 80-84 | 0.55 | 0.55 | 0.55 | 0.63 | 0.63 | 0.63 |
| 85+ | 0.65 | 0.65 | 0.65 | 0.79 | 0.79 | 0.79 |
| **Cancer of the Pancreas*** | | | | | | |
|  | Males | | | Females | | |
| Age* | Never | Current | Former | Never | Current | Former |
| 35-39 | 1.00 | 1.00 | 1.00 | 0.80 | 0.80 | 0.80 |
| 40-44 | 0.61 | 0.61 | 0.61 | 0.33 | 0.33 | 0.33 |
| 45-49 | 1.00 | 1.00 | 1.00 | 0.77 | 0.77 | 0.77 |
| 50-54 | 0.52 | 0.52 | 0.52 | 0.42 | 0.42 | 0.42 |
| 55-59 | 1.00 | 1.00 | 1.00 | 1.00 | 1.00 | 1.00 |
| 60-64 | 0.62 | 0.62 | 0.62 | 0.62 | 0.62 | 0.62 |
| 65-69 | 1.00 | 1.00 | 1.00 | 0.97 | 0.97 | 0.97 |
| 70-74 | 0.83 | 0.83 | 0.83 | 0.69 | 0.69 | 0.69 |
| 75-79 | 0.98 | 0.98 | 0.98 | 0.90 | 0.90 | 0.90 |
| 80-84 | 0.81 | 0.81 | 0.81 | 0.75 | 0.75 | 0.75 |
| 85+ | 1.00 | 1.00 | 1.00 | 1.00 | 1.00 | 1.00 |
|  |  |  |  |  |  |  |
|  |  |  |  |  |  |  |
| **Probability of disease-specific mortality by age, sex, and smoking status, conditional on having a disease event (Continued)** | | | | | | |
| **Cancer of the Larynx*** | | | | | | |
|  | Males | | | Females | | |
| Age* | Never | Current | Former | Never | Current | Former |
| 35-39 | - | - | - | - | - | - |
| 40-44 | - | - | - | - | - | - |
| 45-49 | 0.45 | 0.45 | 0.45 | - | - | - |
| 50-54 | 0.20 | 0.20 | 0.20 | - | - | - |
| 55-59 | 0.35 | 0.35 | 0.35 | - | - | - |
| 60-64 | 0.22 | 0.22 | 0.22 | - | - | - |
| 65-69 | 0.37 | 0.37 | 0.37 | - | - | - |
| 70-74 | 0.31 | 0.31 | 0.31 | - | - | - |
| 75-79 | 0.51 | 0.51 | 0.51 | - | - | - |
| 80-84 | 0.53 | 0.53 | 0.53 | - | - | - |
| 85+ | 0.61 | 0.61 | 0.61 | - | - | - |
| **Cancer of the Lung and Bronchus*** | | | | | | |
|  | Males | | | Females | | |
| Age* | Never | Current | Former | Never | Current | Former |
| 35-39 | 1.00 | 1.00 | 1.00 | 1.00 | 1.00 | 1.00 |
| 40-44 | 0.76 | 0.76 | 0.76 | 0.69 | 0.69 | 0.69 |
| 45-49 | 1.00 | 1.00 | 1.00 | 1.00 | 1.00 | 1.00 |
| 50-54 | 0.58 | 0.58 | 0.58 | 0.49 | 0.49 | 0.49 |
| 55-59 | 1.00 | 1.00 | 1.00 | 1.00 | 1.00 | 1.00 |
| 60-64 | 0.80 | 0.80 | 0.80 | 0.79 | 0.79 | 0.79 |
| 65-69 | 1.00 | 1.00 | 1.00 | 0.87 | 0.87 | 0.87 |
| 70-74 | 0.85 | 0.85 | 0.85 | 0.63 | 0.63 | 0.63 |
| 75-79 | 0.93 | 0.93 | 0.93 | 0.56 | 0.56 | 0.56 |
| 80-84 | 0.85 | 0.85 | 0.85 | 0.57 | 0.57 | 0.57 |
| 85+ | 0.86 | 0.86 | 0.86 | 0.59 | 0.59 | 0.59 |
| **Cancer of the Cervix Uteri*** | | | | | | |
|  | Males | | | Females | | |
| Age* | Never | Current | Former | Never | Current | Former |
| 35-39 | - | - | - | 0.14 | 0.14 | 0.14 |
| 40-44 | - | - | - | 0.13 | 0.13 | 0.13 |
| 45-49 | - | - | - | 0.21 | 0.21 | 0.21 |
| 50-54 | - | - | - | 0.23 | 0.23 | 0.23 |
| 55-59 | - | - | - | 0.47 | 0.47 | 0.47 |
| 60-64 | - | - | - | 0.45 | 0.45 | 0.45 |
| 65-69 | - | - | - | 0.45 | 0.45 | 0.45 |
| 70-74 | - | - | - | 0.46 | 0.46 | 0.46 |
| 75-79 | - | - | - | 0.48 | 0.48 | 0.48 |
| 80-84 | - | - | - | 0.50 | 0.50 | 0.50 |
| 85+ | - | - | - | 0.73 | 0.73 | 0.73 |
|  |  |  |  |  |  |  |
|  |  |  |  |  |  |  |
| **Probability of disease-specific mortality by age, sex, and smoking status, conditional on having a disease event (Continued)** | | | | | | |
| **Cancer of the Urinary Bladder (Invasive and In Situ)*** | | | | | | |
|  | Males | | | Females | | |
| Age* | Never | Current | Former | Never | Current | Former |
| 35-39 | - | - | - | - | - | - |
| 40-44 | - | - | - | - | - | - |
| 45-49 | 0.16 | 0.16 | 0.16 | - | - | - |
| 50-54 | 0.08 | 0.08 | 0.08 | - | - | - |
| 55-59 | 0.17 | 0.17 | 0.17 | - | - | - |
| 60-64 | 0.09 | 0.09 | 0.09 | - | - | - |
| 65-69 | 0.22 | 0.22 | 0.22 | 0.26 | 0.26 | 0.26 |
| 70-74 | 0.15 | 0.15 | 0.15 | 0.19 | 0.19 | 0.19 |
| 75-79 | 0.24 | 0.24 | 0.24 | 0.32 | 0.32 | 0.32 |
| 80-84 | 0.19 | 0.19 | 0.19 | 0.27 | 0.27 | 0.27 |
| 85+ | 0.44 | 0.44 | 0.44 | 0.58 | 0.58 | 0.58 |
| **Cancer of the Kidney and Renal Pelvis*** | | | | | | |
|  | Males | | | Females | | |
| Age* | Never | Current | Former | Never | Current | Former |
| 35-39 | 0.13 | 0.13 | 0.13 | - | - | - |
| 40-44 | 0.07 | 0.07 | 0.07 | - | - | - |
| 45-49 | 0.20 | 0.20 | 0.20 | 0.28 | 0.28 | 0.28 |
| 50-54 | 0.13 | 0.13 | 0.13 | 0.19 | 0.19 | 0.19 |
| 55-59 | 0.33 | 0.33 | 0.33 | 0.27 | 0.27 | 0.27 |
| 60-64 | 0.24 | 0.24 | 0.24 | 0.20 | 0.20 | 0.20 |
| 65-69 | 0.31 | 0.31 | 0.31 | 0.32 | 0.32 | 0.32 |
| 70-74 | 0.27 | 0.27 | 0.27 | 0.28 | 0.28 | 0.28 |
| 75-79 | 0.43 | 0.43 | 0.43 | 0.43 | 0.43 | 0.43 |
| 80-84 | 0.44 | 0.44 | 0.44 | 0.45 | 0.45 | 0.45 |
| 85+ | 0.62 | 0.62 | 0.62 | 0.82 | 0.82 | 0.82 |
| **Acute Myeloid Leukemia*** | | | | | | |
|  | Males | | | Females | | |
| Age* | Never | Current | Former | Never | Current | Former |
| 35-39 | 0.59 | 0.59 | 0.59 | - | - | - |
| 40-44 | 0.50 | 0.50 | 0.50 | - | - | - |
| 45-49 | 0.63 | 0.63 | 0.63 | 0.60 | 0.60 | 0.60 |
| 50-54 | 0.41 | 0.41 | 0.41 | 0.47 | 0.47 | 0.47 |
| 55-59 | 0.49 | 0.49 | 0.49 | 1.00 | 1.00 | 1.00 |
| 60-64 | 0.28 | 0.28 | 0.28 | 0.72 | 0.72 | 0.72 |
| 65-69 | 1.00 | 1.00 | 1.00 | 0.77 | 0.77 | 0.77 |
| 70-74 | 0.69 | 0.69 | 0.69 | 0.54 | 0.54 | 0.54 |
| 75-79 | 0.93 | 0.93 | 0.93 | 0.79 | 0.79 | 0.79 |
| 80-84 | 0.74 | 0.74 | 0.74 | 0.64 | 0.64 | 0.64 |
| 85+ | 1.00 | 1.00 | 1.00 | 1.00 | 1.00 | 1.00 |
|  |  |  |  |  |  |  |
|  |  |  |  |  |  |  |
| **Probability of disease-specific mortality by age, sex, and smoking status, conditional on having a disease event (Continued)** | | | | | | |
| **Ischemic Heart Disease*** | | | | | | |
|  | Males | | | Females | | |
| Age* | Never | Current | Former | Never | Current | Former |
| 35-39 | 0.14 | 0.14 | 0.14 | 0.07 | 0.07 | 0.07 |
| 40-44 | 0.14 | 0.14 | 0.14 | 0.07 | 0.07 | 0.07 |
| 45-49 | 0.13 | 0.13 | 0.13 | 0.08 | 0.08 | 0.08 |
| 50-54 | 0.13 | 0.13 | 0.13 | 0.08 | 0.08 | 0.08 |
| 55-59 | 0.20 | 0.20 | 0.20 | 0.12 | 0.12 | 0.12 |
| 60-64 | 0.20 | 0.20 | 0.20 | 0.12 | 0.12 | 0.12 |
| 65-69 | 0.28 | 0.28 | 0.28 | 0.23 | 0.23 | 0.23 |
| 70-74 | 0.28 | 0.28 | 0.28 | 0.23 | 0.23 | 0.23 |
| 75-79 | 0.52 | 0.52 | 0.52 | 0.41 | 0.41 | 0.41 |
| 80-84 | 0.52 | 0.52 | 0.52 | 0.41 | 0.41 | 0.41 |
| 85+ | 1.00 | 1.00 | 1.00 | 0.99 | 0.99 | 0.99 |
| **Other Heart Disease*** | | | | | | |
|  | Males | | | Females | | |
| Age* | Never | Current | Former | Never | Current | Former |
| 35-39 | 0.04 | 0.04 | 0.04 | 0.03 | 0.03 | 0.03 |
| 40-44 | 0.04 | 0.04 | 0.04 | 0.03 | 0.03 | 0.03 |
| 45-49 | 0.03 | 0.03 | 0.03 | 0.03 | 0.03 | 0.03 |
| 50-54 | 0.03 | 0.03 | 0.03 | 0.03 | 0.03 | 0.03 |
| 55-59 | 0.05 | 0.05 | 0.05 | 0.04 | 0.04 | 0.04 |
| 60-64 | 0.05 | 0.05 | 0.05 | 0.04 | 0.04 | 0.04 |
| 65-69 | 0.07 | 0.07 | 0.07 | 0.05 | 0.05 | 0.05 |
| 70-74 | 0.07 | 0.07 | 0.07 | 0.05 | 0.05 | 0.05 |
| 75-79 | 0.09 | 0.09 | 0.09 | 0.08 | 0.08 | 0.08 |
| 80-84 | 0.09 | 0.09 | 0.09 | 0.08 | 0.08 | 0.08 |
| 85+ | 0.20 | 0.20 | 0.20 | 0.20 | 0.20 | 0.20 |
| **Cerobrovascular Disease*** | | | | | | |
|  | Males | | | Females | | |
| Age* | Never | Current | Former | Never | Current | Former |
| 35-39 | 0.04 | 0.04 | 0.04 | 0.05 | 0.05 | 0.05 |
| 40-44 | 0.04 | 0.04 | 0.04 | 0.05 | 0.05 | 0.05 |
| 45-49 | 0.05 | 0.05 | 0.05 | 0.05 | 0.05 | 0.05 |
| 50-54 | 0.05 | 0.05 | 0.05 | 0.05 | 0.05 | 0.05 |
| 55-59 | 0.05 | 0.05 | 0.05 | 0.07 | 0.07 | 0.07 |
| 60-64 | 0.05 | 0.05 | 0.05 | 0.07 | 0.07 | 0.07 |
| 65-69 | 0.11 | 0.11 | 0.11 | 0.10 | 0.10 | 0.10 |
| 70-74 | 0.11 | 0.11 | 0.11 | 0.10 | 0.10 | 0.10 |
| 75-79 | 0.24 | 0.24 | 0.24 | 0.24 | 0.24 | 0.24 |
| 80-84 | 0.24 | 0.24 | 0.24 | 0.24 | 0.24 | 0.24 |
| 85+ | 0.69 | 0.69 | 0.69 | 0.51 | 0.51 | 0.51 |
|  |  |  |  |  |  |  |
|  |  |  |  |  |  |  |
| **Probability of disease-specific mortality by age, sex, and smoking status, conditional on having a disease event (Continued)** | | | | | | |
| **Other Vascular Diseases*** | | | | | | |
|  | Males | | | Females | | |
| Age* | Never | Current | Former | Never | Current | Former |
| 35-39 | 0.04 | 0.04 | 0.04 | 0.02 | 0.02 | 0.02 |
| 40-44 | 0.04 | 0.04 | 0.04 | 0.02 | 0.02 | 0.02 |
| 45-49 | 0.05 | 0.05 | 0.05 | 0.04 | 0.04 | 0.04 |
| 50-54 | 0.05 | 0.05 | 0.05 | 0.04 | 0.04 | 0.04 |
| 55-59 | 0.09 | 0.09 | 0.09 | 0.06 | 0.06 | 0.06 |
| 60-64 | 0.09 | 0.09 | 0.09 | 0.06 | 0.06 | 0.06 |
| 65-69 | 0.12 | 0.12 | 0.12 | 0.17 | 0.17 | 0.17 |
| 70-74 | 0.12 | 0.12 | 0.12 | 0.17 | 0.17 | 0.17 |
| 75-79 | 0.24 | 0.24 | 0.24 | 0.22 | 0.22 | 0.22 |
| 80-84 | 0.24 | 0.24 | 0.24 | 0.22 | 0.22 | 0.22 |
| 85+ | 0.52 | 0.52 | 0.52 | 0.63 | 0.63 | 0.63 |
| **Diabetes*** | | | | | | |
|  | Males | | | Females | | |
| Age* | Never | Current | Former | Never | Current | Former |
| 35-39 | 0.03 | 0.03 | 0.03 | 0.02 | 0.02 | 0.02 |
| 40-44 | 0.03 | 0.03 | 0.03 | 0.02 | 0.02 | 0.02 |
| 45-49 | 0.04 | 0.04 | 0.04 | 0.03 | 0.03 | 0.03 |
| 50-54 | 0.04 | 0.04 | 0.04 | 0.03 | 0.03 | 0.03 |
| 55-59 | 0.09 | 0.09 | 0.09 | 0.07 | 0.07 | 0.07 |
| 60-64 | 0.09 | 0.09 | 0.09 | 0.07 | 0.07 | 0.07 |
| 65-69 | 0.21 | 0.21 | 0.21 | 0.19 | 0.19 | 0.19 |
| 70-74 | 0.21 | 0.21 | 0.21 | 0.19 | 0.19 | 0.19 |
| 75-79 | 0.33 | 0.33 | 0.33 | 0.31 | 0.31 | 0.31 |
| 80-84 | 0.33 | 0.33 | 0.33 | 0.31 | 0.31 | 0.31 |
| 85+ | 0.65 | 0.65 | 0.65 | 0.45 | 0.45 | 0.45 |
| **Influenza, Pneumonia*** | | | | | | |
|  | Males | | | Females | | |
| Age* | Never | Current | Former | Never | Current | Former |
| 35-39 | 0.02 | 0.02 | 0.02 | 0.01 | 0.01 | 0.01 |
| 40-44 | 0.02 | 0.02 | 0.02 | 0.01 | 0.01 | 0.01 |
| 45-49 | 0.02 | 0.02 | 0.02 | 0.01 | 0.01 | 0.01 |
| 50-54 | 0.02 | 0.02 | 0.02 | 0.01 | 0.01 | 0.01 |
| 55-59 | 0.02 | 0.02 | 0.02 | 0.02 | 0.02 | 0.02 |
| 60-64 | 0.02 | 0.02 | 0.02 | 0.02 | 0.02 | 0.02 |
| 65-69 | 0.05 | 0.05 | 0.05 | 0.03 | 0.03 | 0.03 |
| 70-74 | 0.05 | 0.05 | 0.05 | 0.03 | 0.03 | 0.03 |
| 75-79 | 0.13 | 0.13 | 0.13 | 0.08 | 0.08 | 0.08 |
| 80-84 | 0.13 | 0.13 | 0.13 | 0.08 | 0.08 | 0.08 |
| 85+ | 0.25 | 0.25 | 0.25 | 0.27 | 0.27 | 0.27 |
|  |  |  |  |  |  |  |
|  |  |  |  |  |  |  |
| **Probability of disease-specific mortality by age, sex, and smoking status, conditional on having a disease event (Continued)** | | | | | | |
| **Chronic Obstructive Pulmonary Disease*** | | | | | | |
|  | Males | | | Females | | |
| Age* | Never | Current | Former | Never | Current | Former |
| 35-39 | - | - | - | - | - | - |
| 40-44 | - | - | - | - | - | - |
| 45-49 | 0.03 | 0.03 | 0.03 | 0.02 | 0.02 | 0.02 |
| 50-54 | 0.03 | 0.03 | 0.03 | 0.02 | 0.02 | 0.02 |
| 55-59 | 0.09 | 0.09 | 0.09 | 0.07 | 0.07 | 0.07 |
| 60-64 | 0.09 | 0.09 | 0.09 | 0.07 | 0.07 | 0.07 |
| 65-69 | 0.03 | 0.37 | 0.16 | 0.01 | 0.27 | 0.15 |
| 70-74 | 0.03 | 0.39 | 0.17 | 0.02 | 0.35 | 0.19 |
| 75-79 | 0.07 | 0.95 | 0.31 | 0.05 | 0.56 | 0.32 |
| 80-84 | 0.07 | 1.05 | 0.34 | 0.07 | 0.73 | 0.42 |
| 85+ | 0.12 | 1.66 | 0.54 | 0.08 | 0.79 | 0.45 |

*IMPORTANT: The granular age strata and disease categories shown in this table are used in the model to obtain more precise estimates of life years and quality adjusted life years. However, the rates for an individual age group or disease category may be inaccurate by themselves because the underlying relative risks by smoking status are based on broader age groups and combined disease categories. Some of the estimates shown in this table are obtained by applying a relative risk for a broader age group and combined disease categories to more granular age groups and disease categories encompassed by the relevant relative risk estimate. For this reason, model results are reported only for broader categories and the estimates shown in this table should not be used or reported by themselves. For the age strata and disease groups on which the relative risks are estimated, please see U.S. Department of Health and Human Services. The Health Consequences of Smoking—50 Years of Progress: A Report of the Surgeon General, 2014. Atlanta, GA: U.S. Department of Health and Human Services, Centers for Disease Control and Prevention, National Center for Chronic Disease Prevention and Health Promotion, Office on Smoking and Health; 2014.
