# Supplementary material for: The 20-year impact of tobacco price and tobacco control expenditure increases in Minnesota, 1998-2017
Source: PLoS One. 2020 Mar 18;15(3):e0230364. doi: 10.1371/journal.pone.0230364 (PMC7080278; doi:10.1371/journal.pone.0230364)
Supplement: S2 Supplement — (DOCX) [file pone.0230364.s002.docx]

**Supplement S2**

HealthPartners Institute
 ModelHealth^TM^: Tobacco MN

Background and Model Implementation of Policies for Minnesota Tobacco Policy Analyses

**September 26, 2019**

Contents

[Tobacco Taxes 2](#_Toc21266409)

[Background 2](#_Toc21266410)

[Approach 2](#_Toc21266411)

[Approach of similar models 2](#_Toc21266412)

[ModelHealth:Tobacco approach 3](#_Toc21266413)

[Literature review 3](#_Toc21266414)

[Derivation of formulas for applying elasticities to changes in smoking status and smoking intensity 5](#_Toc21266415)

[One-time impact on prevalence operating through a temporary change in cessation 5](#_Toc21266416)

[Adjustment to avoid double-counting long-term relapse in the simulation 6](#_Toc21266417)

[On-going initiation impact 7](#_Toc21266418)

[Smoking intensity 8](#_Toc21266419)

[Increasing Investments in Tobacco Control 9](#_Toc21266420)

[Background 9](#_Toc21266421)

[Literature and effect size 9](#_Toc21266422)

[Expenditure elasticities and smoking status 10](#_Toc21266423)

[Tobacco control expenditures and smoking intensity 11](#_Toc21266424)

[Calculation of cumulative expenditures 12](#_Toc21266425)

[Derivation of formulas for applying elasticities to changes in smoking status 14](#_Toc21266426)

[Impact on prevalence operating through a temporary change in cessation 14](#_Toc21266427)

[Adjustment to avoid double-counting long-term relapse in the simulation 15](#_Toc21266428)

[On-going initiation impact 16](#_Toc21266429)

[References 18](#_Toc21266430)

# Tobacco Taxes

## Background

There is an extensive literature on the effect of tobacco taxes. Most of the literature expresses results in terms of price elasticities: the percentage change in a smoking behavior that occurs with each percent change in price. The most common behavior measured is smoking prevalence, though some measures of initiation and cessation have been reported. Tax elasticities have also been reported (the percent change in smoking behavior that occurs with each percent change in *tax)*, but the vast majority of estimates are price elasticities. Studies have also estimated smoking-intensity elasticities - the percentage change in cigarettes smoked among smokers who continue to smoke. Estimates of the effect of taxation that are based on elasticities typically use price elasticities and assume that 100% of the tax increase is passed on to consumers as an equal increase in price. The literature has explored several topics, many without clear conclusion, including whether or not elasticities differ by SES and age, the impact of smuggling to avoid taxes, whether or not elasticities have decreased over time, and the distributional impact of tobacco taxes. The International Agency for Research on Cancer (IARC) published an extensive review of the impact of tobacco taxes in 2011^1^ and the Guide to Community Preventive Services updated the IRAC’s review of price elasticities in 2014.^2^

## Approach

### Approach of similar models

Price elasticities are most frequently measured with smoking prevalence as an outcome. Prevalence is determined by the underlying propensities to initiate, quit and resume smoking. In some models, the effects of a policy change can be approximated through a one-time change in smoking prevalence. However, in a microsimulation model such as the HealthPartners Institute’s ModelHealth™:Tobacco, changes to prevalence must occur through changes to cessation or initiation probabilities. We reviewed the approach taken by similar models in implementing tobacco tax increases through price elasticity estimates to identify best practice for estimating tax impacts. We identified three simulation models that implemented tobacco tax impacts through initiation and cessation transitions rather than through direct change in prevalence: SimSmoke as described in Levy et al.;^3^ the RIVM chronic disease model (CDM) as described by van Baal et al.;^4^ and the Congressional Budget Office’s (CBO) analysis of a federal tax increase.^5^ The approaches used by these three models to estimate the impact on taxes through price elasticities are summarized in Table S2.1.

| **Table S2.1. Approach for modifying initial and ongoing effects of a tobacco tax increase in published analyses** | | | | |
| --- | --- | --- | --- | --- |
| **Study** | **Modeled impact of tax increase through smoking behavior? (Yes/no)** | | | |
|  | **Initial prevalence^a^** | **Ongoing initiation** | **Ongoing cessation** | **Relapse** |
| SimSmoke (Levy et al. 2000) | Yes | Yes | Yes | no |
| CDM (van Baal et al. 2007) | Yes | no | no | no |
| CBO 2012 | Yes | Yes | no | no |
| ^a^Modeled either directly or through an initial impact on adult cessation | | | | |

Models have assumed that the tax impact on prevalence among youth and young adults operates through either initiation or ‘net initiation’. The tax impact on older adults operates through cessation. Each of the three models summarized in Table $S2.$1 includes an initial change in prevalence operating through cessation at the time of the tax increase. Levy et al also modeled an ongoing effect on cessation. Unlike the other models, Van Baal et al. excluded an ongoing impact on initiation while noting that this is a conservative assumption. None of the models assumed an impact of taxes on relapse.

### ModelHealth:Tobacco approach

We follow the CBO model approach of implementing a one-time change in the cessation rate for adults ages 25 and older, and a permanent change in the initiation rate. The change in initiation rate is applied to non-smokers ages 9-25. Modeling a permanent impact of a tax increase on the probability of smoking initiation accounts for the impact of the tax over time as new birth cohorts age into the years of increasing risk for tobacco use.

We also modeled the impact of the tax on the number of cigarettes smoked per day among continuing smokers using smoking intensity elasticities from the literature. The model does not assign disease risks that vary with smoking intensity. However, reducing cigarettes smoked per day in a manner consistent with the evidence is necessary to accurately estimate tax revenues generated by a tax increase.

### Literature review

We reviewed price elasticity estimates that were included in the systematic review reported in IARC Handbooks of Cancer Prevention, Volume 14.^1^ Among the cited US studies, we considered those based on person-level data because person-level studies use more recent data and more rigorous methods. We included one additional US-based person-level study identified in the Community Guide’s supplemental search to the IARC review.^2^ We calculated average elasticity estimates across included studies. When multiple studies used the same data source (such as the National Health Interview Survey) and with largely overlapping data years, we selected one study to represent that data source to prevent one data source from heavily influencing our average elasticity estimates. Typically we chose the study that included the most data years or the most recent study. A second study from the same data source was considered if analysis method and results were substantially different from other studies using that source.

We separately considered estimates for youth^6-17^ and adults.^9,18-23^ Among adults, we studied the variation in elasticity by age group and found compelling evidence that adults younger than age 25 were more responsive to cigarette price changes than older adults. While some evidence indicates that middle-aged adults respond differently to price changes than older adults, the evidence was limited and not consistent and therefore we did not further stratify elasticities by age group. The resulting estimates of elasticities and their use in the simulation model is summarized in the Table $S2.$2. The reported estimates for ages 25+ are based on studies that reported results by age group^9,18-20^, allowing us to derive an estimate that excludes younger adults. We explored an estimate using a different set of studies that included adults of any age.^18,21-23^ The resulting estimate for prevalence elasticity (-0.21) was consistent with the expectation of greater price responsiveness when including younger adults, but the resulting estimate for smoking intensity elasticity (-0.11) was lower than the estimate based on studies excluding younger adults.

| **Table S2.2. Elasticities with respect to tobacco price used in ModelHealth:Tobacco** | | | | |
| --- | --- | --- | --- | --- |
|  | **Prevalence Elasticity** | | **Intensity Elasticity** | |
| **Age group** | **Estimate** | **Use in model** | **Estimate** | **Use in model** |
| **9-17** | -0.63 | Permanent change in initiation | -0.42 | Modify quantity of cigarettes smoked among continuing smokers |
| **18-24** | -0.28 | Permanent change in initiation | -0.26 |  |
| **25+** | -0.16 | One-time change in prevalence, operating as a temporary change in cessation rate | -0.17 |  |

For use in a microsimulation model, we algebraically derived an expression to implement a one-time change in prevalence through a temporary increase in cessation rate: C'_t,i_ = 1+ δ_t,0_ (C_t,i_ -1), where C'_t,i_ is the temporary cessation rate for individual *i* reflecting a price change due to tax, C_t,i_ is the baseline cessation rate for individual *i*, and δ_t,0_ is the operator through which prevalence elasticity (E) impacts cessation and is defined following Levy et al. to be equal to [1+E(P_t_-P_O_))/(P_t_+P_O_)] / [1-E(P_t_-P_O_))/(P_t_+P_O_)] in which P_t_ and P_O_ are prices in period *t* and the base year respectively. The algebraic derivation is provided below, where we also note an adjustment to account for relapse among those who quit due to the price increase. Also as shown below is the algebraic derivation of the tax impact on initiation rate as implemented by Levy et al.: I'_t,i_ = δ_t,0_ I_o_.

The impact of a price change on smoking intensity among continuing smokers is obtained directly from the definition of price elasticity as shown below: Q_t,i_' = δ_t,0_ Q_o_, where Q_o_ and Q_t,i_' represent the quantity of cigarettes smoked by individual *i* per day pre- and post-price increase.

## Derivation of formulas for applying elasticities to changes in smoking status and smoking intensity

In all derivations below, we omit subscript *i* to represent individual probabilities of smoking behavior change that vary with demographic characteristics in the microsimulation model. When implemented in the model, the same relative impact of a tax increase is applied to all modeled individuals.

### One-time impact on prevalence operating through a temporary change in cessation

Let C_t_' be the cessation probability with a price increase in period *t*, C_t_ the baseline cessation probability (without price increase), and α be the cessation probability attributable to a price increase. Presuming for the moment that there is no relapse, then

$$C_{t}^{'}= C_{t}+{(1-C}_{t})\times\alpha[Equation S2.1]$$

That is, the cessation probability immediately following a price increase will be equal to the baseline cessation probability plus the cessation impact of the tax among those who would not have quit given the baseline cessation probability alone.

Following Levy et al.^3^, Price Elasticity (E) can be expressed as:

$$E={\frac{Q_{t}-Q_{o}}{Q_{t}+Q_{o}}}/{\frac{P_{t}-P_{o}}{P_{t}+P_{o}}} [Equation S2.2]$$

where the Quantity (Q) and Price (P) have subscripts indicated in their initial (o) and current (t) levels. Note that in calculating elasticities, the denominator in each percentage change is the average of the old and new levels (i.e. (Q_t_ – Q_o_)/[(Q_t_+Q_o_)/2] and (P_t_ – P_o_)/[(P_t_+P_o_)/2] where the 2’s in the percentage change for quantity and price factor-out in the equation for elasticity).

Solving the elasticity formula for Q_t_ yields,

$$Q_{t}=Q_{o}\times\left\{ \frac{1+E\left( \frac{P_{t}-P_{o}}{P_{t}+P_{o}} \right)}{1-E\left( \frac{P_{t}-P_{o}}{P_{t}+P_{o}} \right)} \right\} . [Equation S2.3]$$

With Q defined as the quantity of smokers, then it is also the case that

$Q_{t}=Q_{o}\times\left( 1-\alpha\right) . [Equation S2.4$] (i.e. baseline smokers less those who quit with a tax increase)

Solving both equations for Q_t_/Q_0_ and expressing the results of both as an equality yields,

$$\left\{ \frac{1+E\left( \frac{P_{t}-P_{o}}{P_{t}+P_{o}} \right)}{1-E\left( \frac{P_{t}-P_{o}}{P_{t}+P_{o}} \right)} \right\}=1-\alpha. [Equation S2.5]$$

Rearranging,

$$\alpha= 1-\left\{ \frac{1+E\left( \frac{P_{t}-P_{o}}{P_{t}+P_{o}} \right)}{1-E\left( \frac{P_{t}-P_{o}}{P_{t}+P_{o}} \right)} \right\} . [Equation S2.6]$$

Finally, we obtain the one-time change in the cessation rate from a tobacco tax by substituting this expression for α into the first equation above and simplifying the expression to

$$C_{t}^{'}= 1+{\left\{ \frac{1+E\left( \frac{P_{t}-P_{o}}{P_{t}+P_{o}} \right)}{1-E\left( \frac{P_{t}-P_{o}}{P_{t}+P_{o}} \right)} \right\}(C}_{t}-1). [Equation S2.7]$$

### Adjustment to avoid double-counting long-term relapse in the simulation

The elasticities obtained from the literature are measured in cross-sectional data comparing smoking status to cigarette prices in the same year that smoking status was reported. Therefore, the elasticity estimates reflect a mixed duration of relapse risk. For the portion of tax-induced quits that occurred in the year that smoking status was reported, only short-term relapse is reflected in the elasticity. For these quits, the simulation model’s long-term relapse probabilities will be applied appropriately. For the portion of quits that occurred in earlier years (near the time of an earlier tax change), some long term relapse already occurred. Applying the model’s long-term relapse rates to those quits will double-count that relapse. To prevent double counting of relapse, we adjust the cessation rates attributable to a tax increase upward to approximate a short-term cessation rate prior to long-term relapse.

In the microsimulation model, relapse probabilities are lower with each subsequent year after the quit, and the complement of cumulative relapse = (1-R_(t+1))(1-R_(t+2) )…((1-R_(t+6)) evaluates to 0.513. That is, among the quits which are sustained through the end of year in which the quit was reported, 51.3% will not ever relapse and 48.7% will start smoking again sometime within the next 10 years. We do not know what portion of long-term relapse from a tax increase occurred by the time smoking status was obtained for measuring elasticity. Therefore, we employ a rough estimate that assumes, on average, half of eventual relapse had occurred at the time of elasticity measurement. This implies that the complement of remaining cumulative relapse is 0.756 (=0.513 + (1-.513)*0.5). With this assumption, we modify Equation $S2.$1 such that the initial relapse-adjusted cessation rate (CRA'_t_) reflects cessation prior to long-term relapse among the quits attributable to a tax increase as follows:

$${CRA}_{t}^{'}= C_{t}+\frac{\left. \left( C_{t}^{'}-C_{t} \right) \right.}{0.871}. [Equation S2.8]$$

Although imprecise, we feel this adjustment is better than either failing to adjust for the double-counting of relapse that would occur when the model’s relapse rate is applied or making an adjustment that assumes that 100% of long-term relapse attributable to a price change has already occurred at the time smoking status was assessed for the estimation of elasticity.

Equation $S2.$8 is implemented in the model with substitution of the expression for C'_t_ in Equation $S2$.7. As noted above, this is implemented in the model only as a one-time change in cessation and therefore CRA'_t_ is computed only for the first year of a tax increase.

### On-going initiation impact

Similar to the approach for cessation, Let I_t_' be the initiation rate in period *t* after a price increase, I_t_ the baseline initiation rate (without price increase), and β be the initiation reduction attributable to a price increase.

$$I_{t}^{'}= I_{t}\times\left( 1-\beta\right) . [Equation S2.9]$$

With Q_t_ equal to baseline prevalence less the reduction in initiation due to the tax:

$$Q_{t}=Q_{o}\times\left( 1+\beta\right), [Equation S2.10]$$

substituting Q_t_ with the expression from Equation $S2.$3 and solving for β yields

$$\beta=\left\{ \frac{1+E\left( \frac{P_{t}-P_{o}}{P_{t}+P_{o}} \right)}{1-E\left( \frac{P_{t}-P_{o}}{P_{t}+P_{o}} \right)} \right\}-1 . [Equation S2.11]$$

Finally, substituting this expression for β into Equation $S2.$8 and simplifying yields the expression for the initiation rate reported by Levy et al.:

$$I_{t}^{'}= I_{t}\left\{ \frac{1+E\left( \frac{P_{t}-P_{o}}{P_{t}+P_{o}} \right)}{1-E\left( \frac{P_{t}-P_{o}}{P_{t}+P_{o}} \right)} \right\} . [Equation S2.12]$$

### Smoking intensity

The quantity of cigarettes smoked after a change in price is calculated directly by

$$Q_{t}=Q_{o}\times\left\{ \frac{1+E\left( \frac{P_{t}-P_{o}}{P_{t}+P_{o}} \right)}{1-E\left( \frac{P_{t}-P_{o}}{P_{t}+P_{o}} \right)} \right\}$$

where E is the elasticity for intensity of smoking and where, in this case, Q_t_ and Q_o_ are the number of cigarettes smoked per day by each smoker before and after taxes (among continuing smokers after the tax increase).

# Increasing Investments in Tobacco Control

## Background

Comprehensive tobacco control (CTC) programs have been recommended by the Task Force on Community Preventive Services based on evidence that they reduce tobacco use and prevent tobacco-related disease and death.^24^ Tobacco control programs combine multiple strategies in a coordinated effort among multiple partners. The strategies employed may include large- and small-scale media, school-based education, quitlines with access to free smoking cessation medications, decreasing tobacco accessibility to minors, and facilitating local adoption of regulations such as clean air policies. While many of these strategies have been evaluated individually, the synergies between particular strategies are not clear, making it difficult to determine which combination of strategies provides the most cost-effective tobacco control program.

## Literature and effect size

The Community Guide’s evidence review organizes the broad evidence base for CTC programs.^25^ Most studies provide evaluations of individual state tobacco control programs, particularly in California, Massachusetts and New York (including NY City’s program). These studies compare results within the state pre- and post-implementation or compare trends in outcomes within the state to trends in other states for comparison purposes. Other studies compare tobacco control efforts across all states without focusing on any particular state. These studies use tobacco control expenditures or appropriations as a proxy for intensity of tobacco control and assess the association between expenditures and smoking behaviors.

This later group of studies is of particular interest in assessing the potential impact of increasing investments in tobacco control. The reported effect sizes allow extrapolation to investments of varying size. Therefore, the simulation model can use the estimated relationship between expense and tobacco use to estimate the effect of each year’s investment compared to the base year level. In addition, this literature controls for state tobacco prices.

Within these cross-state studies, we focus on studies which provide estimates of expenditure elasticities that we can apply in the simulation model in a manner similar to the price elasticities used to simulate the impact of tobacco taxes. Expenditure elasticities are estimates of the percent change in smoking behavior that occurs with each percent change in tobacco control expenditures. In the literature, expenditure elasticities are generally estimated on cumulative expenditures over current and prior years with the assumption that prior years’ expenditures still impact current year initiation and cessation. However, studies assume that the impact of prior years’ expenditures are less with each successive year and authors have discounted prior years’ expenditures by 10-50% per year when tabulating cumulative expenditures.

### Expenditure elasticities and smoking status

A series of studies by Farrelly et al. use a consistent methodology to estimate the impact of tobacco control expenditures on youth,^26^ young adult^27^ and all adult^28^ smoking status as summarized in Table $S2.$3. For all adults, Farrelly et al. discount prior year’s expenditures by 10%, 25%, and 50%. We used the estimates with 25% discounting because 25% discounting was used in the estimates for youth and young adults and also in the studies on which we base our estimates of the impact of comprehensive tobacco control expenditures on smoking intensity (see below). As with price elasticities for tobacco taxes, we simulate the impact on young adults (ages 18-24) through reduced net initiation. The estimated expenditure elasticities for youth and young adult initiation can be directly applied in the model as described below.

| **Table S2.3. Expenditure elasticities with respect to smoking status used in ModelHealth:Tobacco** | | |
| --- | --- | --- |
| **Age group** | **Estimate** | **Use in model** |
| **9-17** | -0.059 | Change in initiation probability |
| **18-24** | -0.040 | Change in initiation probability |
| **25+** | -0.016 | Change in cessation probability |

For adults 25 years of age and above, the reported elasticities provide the percentage change in smoking prevalence associated with a one percent change in tobacco control expenditures. Simulation models that derive prevalence through changes in initiation and cessation such as ModelHealth: Tobacco MN cannot directly utilize elasticities measured with prevalence as the outcome. For use in the microsimulation model, we algebraically derived an expression to implement a temporary change in prevalence through a temporary increase in cessation rate in the same manner that we implemented price elasticities when estimating the impact of a change in tobacco tax where we followed Levy et al. The expression is C'_t,i_ = 1+ δ_t,0_ (C_t,i_ -1), where C'_t,i_ is the temporary cessation rate for individual *i* reflecting a price change due to tax, C_t,i_ is the baseline cessation rate for individual *i*, and δ_t,0_ is the operator through which prevalence elasticity (E) impacts cessation and is defined as [1+E(CTC_t_-CTC_O_))/(CTC_t_+CTC_O_)] / [1-E(CTC_t_-CTC_O_))/( CTC_t_+CTC_O_)] in which CTC_t_ and CTC_O_ are expenditures on CTC in period *t* and the base year respectively. See below for algebraic derivation, where we also note an adjustment to account for relapse among those who quit due to the expenditure change.

With these formulas, the elasticity estimates summarized above, and the Minnesota average cumulative baseline per capita tobacco control expenditure of **$**1.17 per capita (Table $S2.$4), ModelHealth:Tobacco MN modifies each subsequent years’ cessation and initiation probabilities relative to the 1997 baseline probabilities based upon the percent difference in cumulative expenditures in each year relative to those of the baseline year.

### Tobacco control expenditures and smoking intensity

The Community Guide found that CTC studies report a median decrease of in cigarettes per day among continuing smokers (smoking intensity) of 17.1% (23.7% when limited to U.S. studies). Thus, increasing tobacco control expenditures could reduce sales beyond the reduction of smoking prevalence. However of the 11 studies identified by the Community Guide, only two studies assessed the relationship between the level of tobacco control expenditures and smoking intensity while controlling for differences in cigarette prices.^29,30^ Rhoads estimated the association between adult smoking intensity and tobacco control expenditures measured in 4 ways: cumulative per capita expenditures with discounting of past year expenditures by 10%, 25%, and 50% per year, and current year per capita expenditures.^29^ Applying the coefficient based on cumulative expenditures with 25% discounting (the same measure of expenditures used when estimating the impact of CTC expenditures on smoking initiation and cessation probabilities), yields an estimate of a 1.44% reduction in smoking intensity for every $1.00 increase in per capita cumulative tobacco control expenditures, with past years’ expenditures discounted 25% per year.

Taurus estimated the association between youth smoking intensity and tobacco control expenditures with 3 model specifications that differ with regard to controlling for state-level influences.^30^ Using the average based on the 3 model specifications yields an estimate of a 4.07% reduction in smoking intensity for youth for every $1.00 increase in current CTC expenditures. Taurus et al. did not report estimates based on cumulative expenditures with discounting. As noted above, for adults Rhoads provided estimates for both cumulative expenditures with 25% discounting of past expenditures and current expenditures (or, equivalently, cumulative expenditures with 100% discounting of past years). The ratio of the reduction of smoking intensity in adults with a cumulative expenditures with 25% discounting compared to current expenditures only was 0.629 (=1.44%/2.26%). Appling this ratio to estimate for current expenditures for youth based on the estimates of Taurus et al. yields an approximate estimate of a 2.56% reduction in youth smoking intensity for every $1.00 increase in per cumulative capita CTC expenditures discounted 25% per year (=0.629 x 4.07%).

In the microsimulation model, these estimates are used to reduce the cigarettes smoked per day for youth and adults when investments in tobacco control are increased, providing a more realistic estimate of tax revenues generated when both taxes investments in tobacco control are increased.

## Calculation of cumulative expenditures

Published studies estimate the elasticity based on cumulative expenditures over current and prior years with the assumption that prior years’ expenditures still impact current year initiation and cessation. However studies assume that the impact of prior years’ expenditures are less with each successive year. For consistency with the measures of effect size used in the simulation model, we use a 25% annual discount of the impact of prior years’ expenditures to calculate cumulative expenditures. That is, we measure cumulative expenditures in Year t as the sum of Exp_t_ + Exp_t-1_ × (1-.25) + Exp_t-2_ × (1-.25)^^2^ +…. through year = t-22, matching the length of the historical series presented in the table below. Note that due to availability of historical estimates for more years, we use appropriations as a proxy for expenditures. The calculation is illustrated in Table S2.4.

| **Table S2.4. Cumulative expenditure calculation based on historical appropriations** | | | |
| --- | --- | --- | --- |
| **Model  year** | **Calendar year** | **Undiscounted annual Minnesota per capita appropriations ($2017)** | **Cumulative Minnesota per capita appropriations, discounted 25% annually** |
| **-11** | 1986^a^ | 0.29 |  |
| **-10** | 1987^a^ | 0.29 |  |
| **-9** | 1988^a^ | 0.29 |  |
| **-8** | 1989^a^ | 0.29 |  |
| **-7** | 1990^a^ | 0.29 |  |
| **-6** | 1991 | 0.32 |  |
| **-5** | 1992 | 0.27 |  |
| **-4** | 1993 | 0.44 |  |
| **-3** | 1994 | 0.41 |  |
| **-2** | 1995 | 0.59 |  |
| **-1** | 1996 | 0.47 |  |
| **0  (model baseline)** | **1997** | **0.45** | **1.71** |
| **1** | 1998 | 0.68 | 1.96 |
| **2** | 1999 | 0.17 | 1.64 |
| **3** | 2000 | 2.63 | 3.85 |
| **4** | 2001 | 5.50 | 8.38 |
| **5** | 2002 | 4.58 | 10.87 |
| **6** | 2003 | 4.97 | 13.12 |
| **7** | 2004 | 3.09 | 12.92 |
| **8** | 2005 | 2.70 | 12.39 |
| **9** | 2006 | 3.07 | 12.36 |
| **10** | 2007 | 2.90 | 12.17 |
| **11** | 2008 | 2.83 | 11.95 |
| **12** | 2009 | 2.58 | 11.54 |
| **13** | 2010 | 2.54 | 11.19 |
| **14** | 2011 | 2.45 | 10.84 |
| **15** | 2012 | 2.41 | 10.54 |
| **16** | 2013 | 2.27 | 10.16 |
| **17** | 2014 | 2.27 | 9.89 |
| **18** | 2015 | 2.27 | 9.68 |
| **19** | 2016 | 2.27 | 9.51 |
| **20** | 2017 | 2.27 | 9.35 |
| ^a^Appropriations are available only from 1991 forward. Prior years are assigned the average of 1991 and 1992 appropriations. Due to discounting, a $.10 error in 1990 appropriations would contribute only $0.0046 in error to 1997 discounted cumulative expenditures. | | | |

## Derivation of formulas for applying elasticities to changes in smoking status

These derivations follow our derivations for applying literature-based price elasticities to estimate the impact of tobacco taxes, which in turn follow Levy et al.^31^

### Impact on prevalence operating through a temporary change in cessation

Let C'_t_ be the cessation rate in period t after an increase in comprehensive tobacco control expenditures, C_t_ the baseline cessation rate (without tobacco control expenditure increase), and $\gamma$ be the cessation rate attributable to a CTC expenditure increase. Presuming for the moment no initiation or relapse, then

$$C_{t}^{'}= C_{t}+{(1-C}_{t})\times\gamma. [Equation S2.13]$$

That is, the cessation rate immediately following a CTC expenditure increase will be equal to the baseline cessation rate plus the cessation rate impact of the tax among those who would not have quit with the baseline cessation rate alone.

Following Levy et al. ^3^, but with different notation for expenditure elasticity rather than price elasticity, CTC expenditure elasticity (E) can be expressed as:

$$E={\frac{Q_{t}-Q_{o}}{Q_{t}+Q_{o}}}/{\frac{{CTC}_{t}-{CTC}_{o}}{{CTC}_{t}+{CTC}_{o}}} [Equation S2.14]$$

where the Quantity (Q) and CTC expenditures (CTC) have subscripts indicated in their original (o) and current (t) levels. Note that in calculating elasticities, the denominator in each percentage change is the average of the old and new levels (i.e. (Q_t_ – Q_o_)/[(Q_t_+Q_o_)/2] where the 2’s in the percentage change for quantity and expenditures factor-out in the equation for elasticity.

Solving the elasticity formula for Q_t_ yields,

$$Q_{t}=Q_{o}\times\left\{ \frac{1+E\left( \frac{{CTC}_{t}-{CTC}_{o}}{{CTC}_{t}+{CTC}_{o}} \right)}{1-E\left( \frac{{CTC}_{t}-{CTC}_{o}}{{CTC}_{t}+{CTC}_{o}} \right)} \right\} . [Equation S2.15]$$

With Q defined as the quantity of smokers, then it is also the case that

$$Q_{t}=Q_{o}\times\left( 1-\gamma\right) . [Equation S2.16]$$

Solving both equations for Q_t_/Q_0_ and expressing the results of both as an equality yields,

$$\left\{ \frac{1+E\left( \frac{{CTC}_{t}-{CTC}_{o}}{{CTC}_{t}+{CTC}_{o}} \right)}{1-E\left( \frac{{CTC}_{t}-{CTC}_{o}}{{CTC}_{t}+{CTC}_{o}} \right)} \right\}=1-\gamma. [Equation S2.17]$$

Rearranging,

$$\gamma= 1-\left\{ \frac{1+E\left( \frac{{CTC}_{t}-{CTC}_{o}}{{CTC}_{t}+{CTC}_{o}} \right)}{1-E\left( \frac{{CTC}_{t}-{CTC}_{o}}{{CTC}_{t}+{CTC}_{o}} \right)} \right\} . [Equation S2.18]$$

Finally, we obtain the one-time change in the cessation rate from a tobacco tax by substituting this expression for $\gamma$ into the first equation above and simplifying the expression to

$$C_{t}^{'}= 1+{\left\{ \frac{1+E\left( \frac{{CTC}_{t}-{CTC}_{o}}{{CTC}_{t}+{CTC}_{o}} \right)}{1-E\left( \frac{{CTC}_{t}-{CTC}_{o}}{{CTC}_{t}+{CTC}_{o}} \right)} \right\}(C}_{t}-1). [Equation S2.19]$$

### Adjustment to avoid double-counting long-term relapse in the simulation

We make an adjustment to avoid double counting of long-term relapse, in the same manner that we did for tobacco tax increases.

The elasticities obtained from the literature are measured in cross-sectional data comparing smoking status to cumulative tobacco control expenditures measured in the same year that smoking status was reported. Therefore, the elasticity estimates reflect a mixed duration of relapse risk. For the portion of CTC-attributable quits that occurred in the year that smoking status was reported, only short-term relapse is reflected in the elasticity. For these quits, the simulation model’s long-term relapse probabilities will be applied appropriately. For the portion of quits that occurred in earlier years, some long term relapse already occurred. Applying the model’s long-term relapse rates to those quits will double-count that relapse. To prevent double counting of relapse, we adjust the cessation rates attributable to increased investments in tobacco control upward to approximate a short-term cessation rate prior to long-term relapse.

In the microsimulation model, relapse probabilities are lower with each year subsequent year after the quit, and the complement of cumulative relapse = (1-R_(t+1))(1-R_(t+2) )…((1-R_(t+6)) evaluates to 0.513. That is, among the quits which are sustained through the end of year in which the quit was reported, 51.3% will not ever relapse and 48.7% will start smoking again sometime within the next 10 years. We do not know what portion of long-term relapse from a tax increase occurred by the time smoking status was obtained for measuring elasticity. Therefore, we employ a rough estimate that assumes, on average, half of eventual relapse had occurred at the time of elasticity measurement. This implies that the complement of remaining cumulative relapse is 0.756 (=0.513 + (1-.513)*0.5). With this assumption, we modify Equation $S2.$13 such that the initial relapse-adjusted cessation rate (CRA'_t_) reflects cessation prior to long-term relapse among the quits attributable to a CTC expenditure increase as follows:

$${CRA}_{t}^{'}= C_{t}+\frac{\left. \left( C_{t}^{'}-C_{t} \right) \right.}{0.871}. [Equation S2.20]$$

Although imprecise, we feel this adjustment is better than either failing to adjust for the double-counting of relapse that would occur when the model’s relapse rate is applied or making an adjustment that assumes that 100% of long-term relapse attributable to a price change has already occurred at the time smoking status was assessed for the estimation of elasticity.

Equation $S2.$20 is implemented in the model with substitution of the expression for C'_t_ in Equation $S2.$19.

### On-going initiation impact

Similar to the approach for cessation, let I_t_' be the initiation rate in period *t* after an increase in CTC expenditure, I_t_ the baseline initiation rate (without expenditure increase), and ε be the initiation rate attributable to an expenditure increase. Presuming for the moment no initiation or relapse, then

$$I_{t}^{'}= I_{t}\times\left( 1-\varepsilon\right) . [Equation S2.21]$$

From

$$Q_{t}=Q_{o}\times\left\{ \frac{1+E\left( \frac{{CTC}_{t}-{CTC}_{o}}{{CTC}_{t}+{CTC}_{o}} \right)}{1-E\left( \frac{{CTC}_{t}-{CTC}_{o}}{{CTC}_{t}+{CTC}_{o}} \right)} \right\} . [Equation S2.22]$$

With Q_t_ defined as the quantity of smokers, then it is also the case that

$$Q_{t}=Q_{o}\times(1+\varepsilon)$$

and hence,

$$\varepsilon=\left\{ \frac{1+E\left( \frac{{CTC}_{t}-{CTC}_{o}}{{CTC}_{t}+{CTC}_{o}} \right)}{1-E\left( \frac{{CTC}_{t}-{CTC}_{o}}{{CTC}_{t}+{CTC}_{o}} \right)} \right\}-1 . [Equation S2.23]$$

Finally, we obtain the change in the initiation rate from a tobacco tax by substituting this expression for ε into the first equation above and simplifying the expression to

$$I_{t}^{'}= I_{o}\left\{ \frac{1+E\left( \frac{{CTC}_{t}-{CTC}_{o}}{{CTC}_{t}+{CTC}_{o}} \right)}{1-E\left( \frac{{CTC}_{t}-{PTC}_{o}}{{CTC}_{t}+{PTC}_{o}} \right)} \right\}. [Equation S2.24]$$

# References

1. IARC Working Group on the Effectiveness of Tax and Price Policies for Tobacco Control. *IARC Handbooks of Cancer Prevention, Tobacco Control, Vol. 14: Effectiveness of Tax and Price Policies for Tobacco Control.* Lyon, France, 2011.

2. Guide to Community Preventive Services. Tobacco Use and Secondhand Smoke Exposure: Interventions to Increase the Unit Price for Tobacco Products. <https://www.thecommunityguide.org/findings/tobacco-use-and-secondhand-smoke-exposure-interventions-increase-unit-price-tobacco-products#GuideReview>. Accessed May 18, 2016.

3. Levy DT, Cummings KM, Hyland A. Increasing taxes as a strategy to reduce cigarette use and deaths: results of a simulation model. *Prev Med.* 2000;31(3):279-286.

4. van Baal PH, Brouwer WB, Hoogenveen RT, Feenstra TL. Increasing tobacco taxes: a cheap tool to increase public health. *Health Policy.* 2007;82(2):142-152.

5. Congressional Budget Office. Raising the Excise Tax on Cigarettes: Effects on Health and the Federal Budget. Washington: Congressional Budget Office; 2012.

6. Lewit EM, Coate D, Grossman M. The effects of government regulation on teenage smoking. *Journal of Law and Economics.* 1981;24:545-569.

7. Lewit EM, Hyland A, Kerrebrock N, Cummings KM. Price, public policy, and smoking in young people. *Tob Control.* 1997;6 Suppl 2:S17-24.

8. Chaloupka FJ, Pacula RL. Sex and race differences in young people's responsiveness to price and tobacco control policies. *Tob Control.* 1999;8(4):373-377.

9. Harris JE, Chan SW. The continuum-of-addiction: cigarette smoking in relation to price among Americans aged 15-29. *Health Econ.* 1999;8(1):81-86.

10. Tauras JAC, F.J. Price, Clean Indoor Air, and Cigarette Smoking: Evidence from the Longitudinal Data for Young Adults. *NBER Working Papers.* 1999;6937.

11. Emery S, White MM, Pierce JP. Does cigarette price influence adolescent experimentation? *J Health Econ.* 2001;20(2):261-270.

12. Gruber JZ, J. Youth Smoking in the United States: Evidence and Implications. In: Gruber J, ed. *Risky Behavior among Youths*. Chicago: Iniversity of Chicago Press; 2001:69-120.

13. Ross H, F.J. C. The Effect of Public Policies and Prices on Youth Smoking. *Southern Economic Journal* 2004;70(4):796-815.

14. DeCicca P, Kenkel D, Mathios A. The fires are not out yet: higher taxes and young adult smoking. *Adv Health Econ Health Serv Res.* 2005;16:293-312.

15. Decicca P, Kenkel D, Mathios A, Shin YJ, Lim JY. Youth smoking, cigarette prices, and anti-smoking sentiment. *Health Econ.* 2008;17(6):733-749.

16. Tauras JA, Markowitz S, Cawley J. Tobacco control policies and youth smoking: evidence from a new era. *Adv Health Econ Health Serv Res.* 2005;16:277-291.

17. Carpenter C, Cook PJ. Cigarette taxes and youth smoking: new evidence from national, state, and local Youth Risk Behavior Surveys. *J Health Econ.* 2008;27(2):287-299.

18. Farrelly MC, Bray JW, Pechacek T, Woollery T. Response by adults to increases in cigarette prices by sociodemographic characteristics. *Southern Economic Journal.* 2001;68(1):156-165.

19. DeCicca P, Kenkel D, Mathios A. Cigarette taxes and the transition from youth to adult smoking: smoking initiation, cessation, and participation. *J Health Econ.* 2008;27(4):904-917.

20. Franz GA. Price effects on the smoking behaviour of adult age groups. *Public Health.* 2008;122(12):1343-1348.

21. Tauras JA. Smoke-free air laws, cigarette prices, and adult cigarette demand. *Economic Inquiry.* 2006;44(2):333–342

22. DeCicca P, McLeod L. Cigarette taxes and older adult smoking: evidence from recent large tax increases. *J Health Econ.* 2008;27(4):918-929.

23. Ong MK, Zhou Q, Sung HY. Sensitivity to cigarette prices among individuals with alcohol, drug, or mental disorders. *Am J Public Health.* 2010;100(7):1243-1245.

24. Guide to Community Preventive Services. Tobacco Use and Secondhand Smoke Exposure: Comprehensive Tobacco Control Programs. 2014; <https://www.thecommunityguide.org/findings/tobacco-use-and-secondhand-smoke-exposure-comprehensive-tobacco-control-programs>. Accessed May 18, 2016.

25. Services GtCP. Reducing Tobacco Use and Secondhand Smoke Exposure: Comprehensive Tobacco Control Programs Summary Evidence Table. In: SET-comprehensive.pdf, ed: Centers for Disease Control and Prevention; 2014.

26. Farrelly MC, Loomis BR, Han B, et al. A comprehensive examination of the influence of state tobacco control programs and policies on youth smoking. *Am J Public Health.* 2013;103(3):549-555.

27. Farrelly MC, Loomis BR, Kuiper N, et al. Are tobacco control policies effective in reducing young adult smoking? *J Adolesc Health.* 2014;54(4):481-486.

28. Farrelly MC, Pechacek TF, Thomas KY, Nelson D. The impact of tobacco control programs on adult smoking. *Am J Public Health.* 2008;98(2):304-309.

29. Rhoads JK. The effect of comprehensive state tobacco control programs on adult cigarette smoking. *J Health Econ.* 2012;31(2):393-405.

30. Tauras JA, Chaloupka FJ, Farrelly MC, et al. State tobacco control spending and youth smoking. *Am J Public Health.* 2005;95(2):338-344.

31. Levy DT, Graham AL, Mabry PL, Abrams DB, Orleans CT. Modeling the impact of smoking-cessation treatment policies on quit rates. *Am J Prev Med.* 2010;38(3 Suppl):S364-372.
