# Supplementary material for: The 20-year impact of tobacco price and tobacco control expenditure increases in Minnesota, 1998-2017
Source: PLoS One. 2020 Mar 18;15(3):e0230364. doi: 10.1371/journal.pone.0230364 (PMC7080278; doi:10.1371/journal.pone.0230364)
Supplement: S3 Supplement — (DOCX) [file pone.0230364.s003.docx]

**Supplement S3**

HealthPartners Institute
 ModelHealth^TM^: Tobacco MN

Annual Result and Supplemental Economic Outcomes Tables

**February 17, 2020**
