# Supplementary material for: The 20-year impact of tobacco price and tobacco control expenditure increases in Minnesota, 1998-2017
Source: PLoS One. 2020 Mar 18;15(3):e0230364. doi: 10.1371/journal.pone.0230364 (PMC7080278; doi:10.1371/journal.pone.0230364)
Supplement: Attachment — Submitted filename: Response to Reviewers.docx [file pone.0230364.s004.docx]

Editor Comments:

There is only one small change left.  In the Introduction, you say that "Large tobacco price increases are the most effective known strategy to reduce tobacco use.”  While this is a commonly made statement, as you note elsewhere in the paper, how effective a tax increase is depends on the size of the tax increase.  For example, a smokefree law could have a bigger effect than a small tax increase.  Please replace "the most effective" with "an effective" and make any other needed wording adjustments to the paper to avoid overstating the importance of tax increases.

**Response:**

**We agree that this is a contradiction. We have made the suggested change in the introduction and removed characterizations of the effect size of tobacco taxes (e.g. ‘large’, ‘substantial’) from the results section.**
